# Conjugation of Synthetic Trisaccharide of *Staphylococcus aureus* Type 8 Capsular Polysaccharide Elicits Antibodies Recognizing Intact Bacterium

**DOI:** 10.3389/fchem.2020.00258

**Published:** 2020-04-28

**Authors:** Ming Zhao, Chunjun Qin, Lingxin Li, Haotian Xie, Beining Ma, Ziru Zhou, Jian Yin, Jing Hu

**Affiliations:** ^1^Key Laboratory of Carbohydrate Chemistry and Biotechnology Ministry of Education, School of Biotechnology, Jiangnan University, Wuxi, China; ^2^Wuxi School of Medicine, Jiangnan University, Wuxi, China

**Keywords:** *Staphylococcus aureus*, capsular polysaccharide, synthetic oligosaccharide, immunogenicity, immunoglobulin G (IgG), monoclonal antibody, carbohydrate-based vaccine

## Abstract

*Staphylococcus aureus* causes a wide range of life-threatening diseases. One of the powerful approaches for prevention and treatment is to develop an efficient vaccine as antibiotic resistance greatly increases. *S. aureus* type 8 capsular polysaccharide (CP8) has shown great potential in vaccine development. An understanding of the immunogenicity of CP8 trisaccharide repeating unit is valuable for epitope-focused vaccine design and cost-efficient vaccine production. We report the chemical synthesis of conjugation-ready CP8 trisaccharide **1** bearing an amine linker, which effectively served for immunological evaluation. The trisaccharide **1**-CRM197 conjugate elicited a robust immunoglobulin G (IgG) immune response in mice. Both serum antibodies and prepared monoclonal antibodies recognized *S. aureus* strain, demonstrating that synthetic trisaccharide **1** can be an efficient antigen for vaccine development.

## Introduction

*Staphylococcus aureus*, one of the most common opportunistic human pathogens, causes a wide range of life-threatening diseases including endocarditis, abscesses, bacteremia, sepsis, and osteomyelitis (Zhang et al., 2018). The high prevalence and antimicrobial resistance of *S. aureus* strains have led to a high incidence of hospital infections and complicated treatment (O'Brien and McLoughlin, 2019). Particularly, the capsular serotype 5 and 8 strains cause most cases of severe disease and death worldwide (Gerlach et al., 2018; Ansari et al., 2019; Mohamed et al., 2019). It is urgent to provide protection against *S. aureus* infections. Vaccines based on isolated bacterial polysaccharides against *Haemophilus influenzae, Neisseria meningitidis*, and *Streptococcus pneumoniae* save millions of lives each year (Broecker et al., 2014). Numerous effort has been made to develop *S. aureus* serotype 5 and 8 capsular polysaccharides (CP5 and CP8)-based vaccine, such as StaphVAX (Nabi Biopharmaceuticals, Rockville, MD) and *S. aureus* four-antigen vaccine (SA4Ag), which show great potential in clinical trials (Shinefield et al., 2002; Fattom et al., 2015; Begier et al., 2017; Creech et al., 2017; Frenck et al., 2017; Ansari et al., 2019; O'Brien and McLoughlin, 2019).

However, the side effects and hyporesponsiveness from impurities and non-protective epitopes have hampered the development of polysaccharide-based vaccine (Anish et al., 2014). Homogeneous polysaccharide antigens after tedious purification steps are required to increase vaccine quality, efficacy, and safety (Anish et al., 2014). Synthetic oligosaccharides provide an attractive alternative to furnish vaccines free of contaminants, particularly against non-culturable pathogens. Tremendous progress has been achieved in the field of developing synthetic oligosaccharide vaccines against human pathogenic bacteria (Verez-Bencomo et al., 2004; Aguilar-Betancourt et al., 2008; Shang et al., 2015; Kong et al., 2016; Liao et al., 2016; Schumann et al., 2017). Synthetic oligosaccharides with well-defined structures can facilitate epitope mapping, which allows for rational epitope design (Broecker et al., 2016). Most polysaccharide chains of pathogens contain repetitive sequences that can be an attractive option for epitope discovery and design (Anish et al., 2014; Schumann et al., 2014; Reinhardt et al., 2015; Menova et al., 2018). The immunogenicity of oligosaccharide antigen can be enhanced and evaluated after conjugation to a carrier protein. Insights into the immunological features of oligosaccharide antigens, such as epitope recognition patterns, binding affinities, and carbohydrate–antibody interactions, can be gained by dissecting oligosaccharide interactions with purified monoclonal antibodies (mAbs) using various biochemical and biophysical techniques (Reinhardt et al., 2015; Broecker et al., 2016; Liao et al., 2016; Emmadi et al., 2017; Lisboa et al., 2017; Kaplonek et al., 2018). Identification of the minimal epitopes of bacterial surface polysaccharides may contribute to more cost-efficient vaccines with limited synthetic effort (Anish et al., 2014; Pereira et al., 2015).

*S. aureus* CP5 and CP8 have been found as highly potent antigenic targets. To date, chemical synthesis of the trisaccharide repeating units of CP5 (Danieli et al., 2012; Yasomanee et al., 2016; Hagen et al., 2017; Behera et al., 2020) and CP8 (Visansirikul et al., 2015) has been achieved. The immunological mechanism remains unclear. During the course of our investigations on the synthesis of complex oligosaccharides, we have successfully completed several complicated bacterial lipopolysaccharide repeating antigens (Qin et al., 2018; Zou et al., 2018; Tian et al., 2020). Here, we describe the design and chemical synthesis of CP8 trisaccharide containing an amine linker at the reducing end with D-glucose and L-fucose as starting materials, which is ready for glycoconjugate preparation and glycan microarray fabrication. The immunogenicity of synthetic trisaccharide was evaluated with glycan microarray after the conjugation with CRM197 protein. The nontoxic diphtheria toxoid mutant CRM197 is often used in licensed vaccines, which can prove highly immunogenic (Hecht et al., 2009; Avci and Kasper, 2010; Broecker et al., 2011). The mAbs were generated and the recognition and binding with *S. aureus* strain were detected, indicating the great potential of synthetic trisaccharide **1** as an efficient vaccine antigen.

## Materials and Methods

### Chemicals and Instruments

Commercially available reagents and solvents (analytical grade) were used without further purification unless otherwise stated. The anhydrous solvents were obtained from an MBraun MB-SPS 800 Dry Solvent System. ^1^H, ^13^C, and two-dimensional NMR spectra were recorded on a Bruker Ultrashield Plus 400 MHz spectrometer at 25°C. High-resolution mass spectra were acquired on an Agilent 6220 ESI-TOF mass spectrometer. Optical rotation (OR) was performed with a Schmidt & Haensch UniPol L 1000 at 589 nm and a concentration (c) expressed in g/100 mL. Infrared (IR) spectra were acquired on Nicolet iS5 spectrometer (Thermo Fisher).

### Synthesis of Trisaccharide 1

**Scheme 1:**
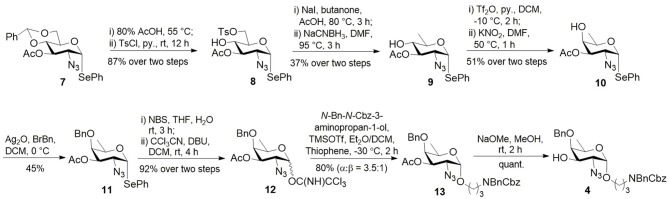
Synthesis of D-fucosamine **4**.

**Scheme 2:**
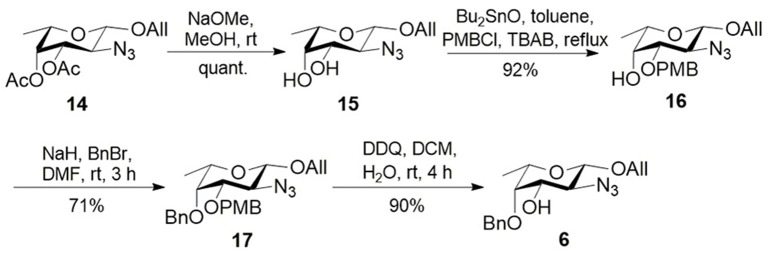
Synthesis of L-fucosamine **6**.

**Scheme 3:**
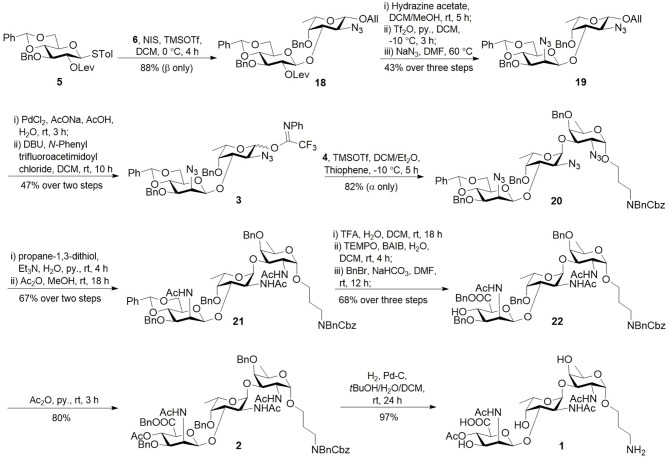
Synthesis of CP8 trisaccharide **1**.

The synthetic route of building blocks **4** and **6** is outlined in Scheme 1, Scheme 2, respectively (synthetic procedure, see Supplementary Material). The synthetic route of target trisaccharide **1** is outlined in Scheme 3.

Procedure for the synthesis of *N*-benzyl-*N*-benzyloxycarbonyl-3-aminopropyl 4-*O*-benzyl-3-*O*-(4-*O*-benzyl-3-*O*-[benzyl 3-*O*-benzyl-2-acetamido-2-deoxy-β-D-mannopyranosyl uronate]-2-acetamido-2-deoxy-α-L-fucopyranosyl)-2-acetamido−2-deoxy-α-D-fucopyranoside (**22**):

To a solution of compound **21** (synthetic procedure, see Supplementary Material) (8.3 mg, 6.7 μmol) in dichloromethane (DCM; 0.7 mL), water (2 μL) and trifluoroacetic acid (70 μL, 0.94 mmol) were added at room temperature. The reaction was stirred overnight and monitored by thin layer chromatography (TLC) analysis. After being quenched with triethylamine (Et_3_N; 0.1 mL), the solvent was evaporated. The residue was purified by silica gel column chromatography (DCM:methanol 20:1 v/v) to give 4,6-diol compound (6 mg, 5.2 μmol, 78%).

Water (0.28 mL), 2,2,6,6-tetramethylpiperidine 1-oxyl (TEMPO) (0.9 mg, 5.5 μmol), and (diacetoxyliodo) benzene (BAIB) (9 mg, 27.7 μmol) were added to a solution of the 4,6-diol compound (12.7 mg, 11 μmol) in DCM (0.6 mL) at room temperature. The mixture was stirred for 4 h and monitored by TLC analysis. After that, the mixture was passed through a pad of silica gel (DCM:methanol 5:1 v/v), concentrated and dried under high vacuum. The crude acid was used in the next step directly.

The crude acid was dissolved in anhydrous N,N-dimethylformamide (DMF; 1.1 mL) under argon. Sodium hydrogen carbonate (5 mg, 50 μmol) and benzyl bromide (BnBr; 10 μL, 82.5 μmol) were added at room temperature. The reaction mixture was stirred at room temperature for 12 h and monitored by TLC analysis. After being quenched with water (2 mL), the reaction mixture was extracted with ethyl acetate (3 mL × 5 mL) and the organic layer was washed with brine (5 mL). The organic layer was dried over Na_2_SO_4_, filtered and concentrated. The residue was purified by silica gel column chromatography (DCM:methanol 50:1 v/v) to afford trisaccharide **22** as a colorless syrup (12.0 mg, 9.6 μmol, 87% over two steps). IR νmax (film) 2,942, 2,865, 1,722, 1,366, 1,294, 1,242, 1,190, 1,104, 1,046, 732 cm^−1^; ^1^H NMR [400 MHz, deuterochloroform (CDCl_3_)] δ = 7.51–7.09 (m, 30H, 5Ph), 6.90 (d, *J* = 9.3 Hz, 1H, N-H), 6.32 (m, 2H, N″-H, N-H), 5.43-5.24 (m, 2H, Bn-2H), 5.13 (s, 2H, Bn-2H), 4.99-4.82 (m, 3H, Bn-2H, 1′-H), 4.83-4.69 (m, 4H, Bn-2H, 2-H, 2′-H), 4.68-4.56 (m, 3H, 1-H, NBn-1H, 2″-H), 4.54 (d, *J* = 2.0 Hz, 1H, 1″-H), 4.47 (dd, *J* = 11.3, 3.4 Hz, 2H, Bn-2H), 4.38 (d, *J* = 15.8 Hz, 1H, NBn-1H), 3.99-3.85 (m, 3H, 5′-H, 3-H, 4″-H), 3.79 (d, *J* = 9.3 Hz, 2H, 5″-H, 5-H), 3.71 (dd, *J* = 10.7, 2.6 Hz, 2H, 3′-H, linker-1H), 3.68-3.57 (m, 1H, linker-1H), 3.50-3.37 (m, 3H, 4-H, 4′-H, 3″-H), 3.28 (s, 1H, linker-1H), 3.20 (d, *J* = 13.9 Hz, 1H, linker-1H), 2.74 (d, *J* = 2.5 Hz, 1H, 4″-OH), 2.05 (s, 3H, CH_3_CO), 2.00 (s, 6H, 2CH_3_CO), 1.71 (m, 2H, linker-2H), 1.26 (d, *J* = 7.0 Hz, 3H, 6′-CH_3_), 1.20 (d, *J* = 6.5 Hz, 3H, 6-CH_3_).

Procedure for the synthesis of *N*-benzyl-*N*-benzyloxycarbonyl-3-aminopropyl 4-*O*-benzyl-3-*O*-(4-*O*-benzyl-3-*O*-[benzyl 4-*O*-acetyl-3-*O*-benzyl-2-acetamido-2-deoxy-β-D-mannopyranosyl uronate]-2-acetamido-2-deoxy-α-L-fucopyranosyl)- 2-acetamido-2-deoxy-α-D- fucopyranoside (**2**):

To a solution of compound **22** (7 mg, 5.6 μmol) in pyridine (0.2 mL) under argon, acetic anhydride (6 μL, 63.5 μmol) was added at 0°C. After stirring for 3 h at room temperature, the reaction was quenched with methanol (50 μL). After removal of solvent, the residue was purified by silica gel column chromatography (DCM:methanol 70:1 v/v) to give product **2** as a colorless syrup (5.8 mg, 4.5 μmol, 80%). ${\left[\alpha \right]}_{\text{D}}^{20}$ = −13.47° [c = 0.50, chloroform (CHCl_3_)]; IR νmax (film) 3,334, 2,923, 1,754, 1,671, 1,526, 1,454, 1,368, 1,232, 1,095, 1,051, 736, 698 cm^−1^; ^1^H NMR (400 MHz, CDCl_3_) δ = 7.51–7.10 (m, 30H, 6Ph), 6.84 (d, *J* = 9.5 Hz, 1H, N-H), 6.57 (d, *J* = 9.1 Hz, 1H, N″-H), 6.36 (d, *J* = 9.6 Hz, 1H, N-H), 5.38 (t, *J* = 6.5 Hz, 1H, 4″-H), 5.27-5.00 (m, 5H, Bn-5H), 4.98 (d, *J* = 3.8 Hz, 1H, 1′-H), 4.91–4.67 (m, 5H, 1-H, 2-H, 2′-H, Bn-2H), 4.67-4.47 (m, 5H, 1″-H, 2″-H, Bn-3H), 4.46-4.34 (m, 2H, Bn-2H), 4.29–4.18 (m, 1H, 3′-H), 4.04 (d, *J* = 6.1 Hz, 1H, 5″-H), 3.96 (m, 3H, 5-H, 5′-H, 3-H), 3.67 (m, 3H, 4-H, linker-2H), 3.51 (m, 2H, 4′-H, 3″-H), 3.38-3.15 (m, 2H, linker-2H), 2.04 (s, 3H, CH_3_CO), 2.00 (s, 3H, CH_3_CO), 1.92 (s, 3H, CH_3_CO), 1.82 (s, 3H, CH_3_CO), 1.76 (m, 2H, linker-2H), 1.25 (d, *J* = 6.4 Hz, 6H, 6-CH_3_, 6′-CH_3_); ^13^C NMR (100 MHz, CDCl_3_) δ = 171.9, 169.7, 169.6, 167.4, 156.4, 139.0, 138.3, 137.9, 136.5, 135.0, 128.8, 128.7, 128.6, 128.5, 128.4, 128.3, 128.2, 128.1, 127.8, 127.5, 127.3, 100.3 (anomeric), 98.0 (anomeric), 94.5 (anomeric), 74.7, 74.4, 72.1, 67.9, 67.7, 67.5, 67.2, 63.5, 49.7, 48.8, 47.7, 47.0, 29.7, 23.6, 22.9, 20.8, 17.3, 17.0; high-resolution electrospray ionization mass spectrometry (HR-ESI-MS) (m/z): calcd for C_72_H_84_N_4_O_18_Na^+^ (M + Na^+^): 1,315.5678, found: 1,315.5697.

Procedure for the synthesis of 3-aminopropyl 3-*O*-(3-*O*-[4-*O*-acetyl-2-acetamido-2-deoxy-β-D-mannopyranosyluronic acid]-2-acetamido-2-deoxy-α-L-fucopyranosyl)-2-acetamido-2-deoxy-α-D-fucopyranoside (**1**):

Trisaccharide **2** (4.7 mg, 3.63 μmol) was dissolved in a mixture of tert-butyl alcohol (*t*BuOH)/water/DCM (5:2:1 v/v/v, 4 mL). The solution was purged with nitrogen, 10% Pd/C was added, and the solution was purged with H_2_ for 5 min, then stirred under an H_2_ atmosphere overnight, filtered (celite pad), and concentrated. The residue was purified with a Sep-Pak cartridge C18 (Macherey-Nagel, Düren, Germany) using water and methanol as eluents to give trisaccharide **1** as a white solid (2.5 mg, 3.53 μmol, 97%). ${\left[\alpha \right]}_{\text{D}}^{20}$ = −45.25° (c = 0.20, H_2_O); ^1^H NMR (400 MHz, D_2_O) δ = 5.13–5.05 (m, 2H, 4″-H, 1′-H), 5.02 (s, 1H, 1″-H), 4.83 (d, *J* = 3.8 Hz, 1H, 1-H), 4.59 (d, *J* = 4.4 Hz, 1H, 2″-H), 4.33 (dd, *J* = 11.1, 3.8 Hz, 1H, 2-H), 4.27–4.20 (m, 2H, 2′-H, 4′-H), 4.13 (dd, *J* = 9.6, 4.5 Hz, 3H, 3″-H, 5-H, 5′-H), 4.08 (s, 1H, 3′-H), 4.04–3.94 (m, 2H, 5″-H, 3-H), 3.85 (d, *J* = 3.2 Hz, 1H, 4-H), 3.81 (dd, *J* = 10.9, 5.6 Hz, 1H, linker-CH_2_), 3.57 (dt, *J* = 11.2, 6.0 Hz, 1H, linker-CH_2_), 3.16 (t, *J* = 7.6 Hz, 2H, linker-CH_2_), 2.18 (s, 3H, CH_3_CO), 2.11 (s, 6H, 2CH_3_CO), 2.03 (s, 5H, linker-CH_2_, CH_3_CO), 1.28 (d, *J* = 2.3 Hz, 3H, 6′-CH_3_), 1.27 (d, *J* = 2.7 Hz, 3H, 6-CH_3_); ^13^C NMR (100 MHz, D_2_O) δ = 175.6, 173.9, 173.8, 173.1, 98.9 (1′-C), 97.2 (1-C), 95.1 (1″-C), 74.1, 73.3, 73.1, 71.1, 70.1, 69.5, 67.6, 66.9, 66.5, 65.0, 53.0, 48.5, 47.6, 37.2, 26.8, 22.3, 21.94, 21.92, 20.3, 15.5, 15.3; HR-ESI-MS (m/z): calcd for C_29_H_48_N_4_O_16_Na^+^ (M + Na^+^): 731.2963, found: 731.2962.

Characterization data: ^1^H, ^13^C, and two-dimensional NMR spectra for products are shown in the Supplementary Material (Pages S14-S33).

### Preparation and Analysis of Glycoconjugate

To a solution of bis(p-nitrophenyl adipate) (PNP; 67.8 μmol) in dimethyl sulfoxide (DMSO)/pyridine (1:1) was added triethylamine (86 μmol) stirred for 5 min at room temperature. Followed by dropwise addition of the compound trisaccharide **1** (2.26 μmol) in a mixture of DMSO and pyridine (1:1) and the reaction mixture was stirred at room temperature for 7 h. The reaction mixture was lyophilized. The solid residue was washed with CHCl_3_. Trisaccharide PNP-ester was obtained. CRM197 protein (0.017 μmol) was washed with autoclaved water and phosphate buffer (pH 8.0). CRM197 in phosphate buffer (100 μL) was added to trisaccharide PNP-ester and stirred at room temperature for 24 h. After the reaction, the mixture was washed with water and phosphate buffer. The glycoconjugate was analyzed by matrix-assisted laser desorption/ionization (MALDI)–time-of-flight (TOF)–MS and sodium dodecyl sulfate (SDS)–polyacrylamide gel electrophoresis (PAGE) analysis.

### Immunization Experiments

Animal experiments were approved by Jiangnan University of Technology Animal Care and Use Committee (Animal Ethics Committee Number: JN.No. 20180915b0121125[175]). Twelve female Balb/c mice (6 weeks old; Charles River, Beijing, China) were randomly divided into two groups. Each mouse of the immunized group was immunized subcutaneously with glycoconjugate corresponding to 4 μg oligosaccharide hapten in complete Freund's adjuvant. The mice were then boosted twice with glycoconjugates in incomplete adjuvants in a 2-week interval. Sham immunized mice were treated with the respective adjuvant without glycoconjugate. Serum samples were drawn from the tail vein and tested every week. According to the mice serum microarray result, the mouse with the highest immunogenicity was boosted once more and euthanized to collect the spleen for cell fusion.

### Preparation of Glycan Microarray Slides and Microarray Binding

Diisopropylamine (DIPA; 3.6 mL) was added into the solution of tetraethylene glycol disuccinimidyl disuccinate (TGDD; 1.58 g) in DMF (257 mL). APTES slides (Electron Microscopy Science) immersed in the solution of TGDD at 40°C with 60–70 rpm, overnight. Then, the slides were sonicated for 15 min and washed with anhydrous ethanol three times. After spin-dry, the slides were vacuum dried at 37°C for 3 h. The oligosaccharides were dissolved in the coupling buffer (50 mM sodium phosphate, pH 8.5) for printing using Arrayjet Sprint (Arrayjet). After printing, the slides were incubated into a humidified chamber at 26°C with 55% humidity overnight. The slides were then placed into microarray quenching buffer (dissolve 50 nM Na_2_HPO_4_ and 100 nM ethanolamine in 1 L ddH_2_O) 50°C for 1 h, then washed with ddH_2_O three times. The slides were shortly centrifuged to remove residual water and then ready for use.

Quenched slides were blocked by incubation in 3% bovine serum albumin (BSA; w/v) in phosphate buffered saline (PBS) at 4°C, overnight. The slides were washed with PBST (0.1% tween in PBS) once, twice with PBS. The residual liquid was removed by centrifugation. Mice serum samples were serially diluted in 1% BSA (w/v) in PBS and then added into the wells of incubation chamber (ProPlate) on the microarray. Each sample has at least two replicates. The microarray was incubated in a dark humid chamber for 1 h at room temperature. The samples were then removed, and each well was washed three times with 50 μL PBST. Secondary antibodies diluted 1:400 in 1% BSA (w/v) in PBS was added into the wells and incubated in the dark humid chamber for 45 min at room temperature. After removing secondary antibodies, each well was washed three times with 50 μL PBST. The chamber was then carefully removed, and the slides were washed once with ddH_2_O and once with ddH_2_O for 15 min. The residual liquid was removed by centrifugation and ready for scanning by Axon GenePix 4200AM.

### Generation and Purification of Monoclonal Antibodies

Isolated splenocytes from the selected mouse were fused to P3X63Ag8.653 myeloma cells (ATCC CRL-1580) with standard hybridoma technique (Broecker et al., 2015). Selection of positive clones specific for trisaccharide **1** was conducted with glycan array screening. Consecutive subcloning steps were performed to identify mAb-producing hybridoma cells. The large-scale production of ascitic fluid rich in mAb was performed according to the published method (Ren et al., 2017), which was approved by Jiangnan University of Technology Animal Care and Use Committee (Animal Ethics Committee Number: JN.No. 20190515b0060831[107]). The mAb was further isolated from ascitic fluid using sequential precipitation with caprylic acid and ammonium sulfate.

### Immunofluorescence of Inactivated *S. aureus*

*S. aureus* serotype 8 (ATCC 49525) and *Escherichia coli* BL21 were cultured in Soybean-Casein Digest Medium and Luria-Bertani (LB) medium at 37°C, respectively. The bacteria were inactivated in 0.4% paraformaldehyde for 48 h. The bacteria were collected and washed with buffer I (50 mM NaHCO_3_, 100 mM NaCl, pH 7.5) for labeling with 0.1 mg·mL^−1^ fluorescein isothiocyanate (FITC). The bacteria were then washed with 0.25% BSA (w/v) in PBS suspended in 1% BSA (w/v) in PBS and incubated with mAbs or mouse serum at 4°C for 16 h under agitation. After being washed with 1% BSA (w/v) in PBS, the bacteria were incubated with goat anti-mouse IgG–Alexa Fluor 635 solution in the dark at room temperature for 1.5 h. After washing, the fluorescence on bacteria was monitored using a confocal laser scanning microscope.

## Results and Discussion

### Synthesis of Trisaccharide 1

**Figure 1:**
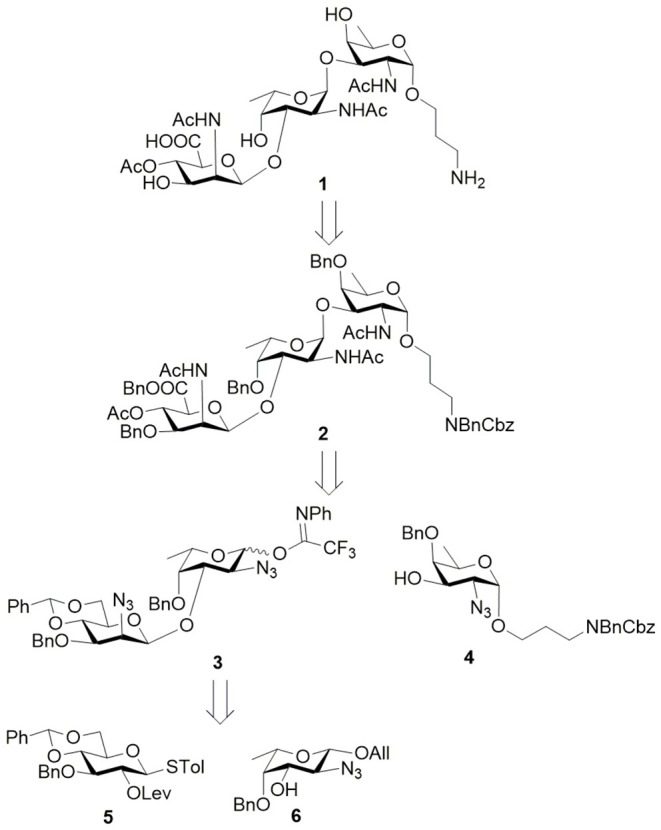
Retrosynthetic analysis of *Staphylococcus aureus* CP8 trisaccharide **1**.

Conjugation-ready *S. aureus* CP8 trisaccharide **1** bearing orthogonal amine linker was designed. Retrosynthetic analysis revealed disaccharide trifluoroacetimidate **3** and D-fucosamine **4** as key intermediates (Figure 1). According to a β-glucosylation–epimerization strategy, disaccharide **3** containing β-mannosidic linkage in turn can be derived from D-glucose building block **5** and L-fucosamine building block **6**. The C2 epimerization was designed to progress at disaccharide stage for improving the overall synthesis efficiency. Particularly, both C2 amino groups in D-fucosamine and L-fucosamine were marked by non-participating azido groups to help the stereoselective formation of two 1,2-*cis*-α-glycosidic linkages.

Preparation of D-fucosamine building block **4** was started from the cheapest D-glucose *via* the known compound **7** (Qin et al., 2018) (Scheme 1). The removal of benzylidene and subsequent tosylation afford compound **8**, which was C6 deoxidized through iodination and reduction in good overall yield. Triflation of D-quinovosamine derivative **9** followed by C4 epimerization through a Lattrell-Dax inversion gave rise to D-fucosamine derivative **10** in 51% overall yield. After benzylation under a neutral condition, compound **11** was converted to Schmidt donor **12** in good overall yield. Acid-catalyzed glycosylation with linker *N*-Bn-*N*-Cbz-3-aminopropan-1-ol in diethyl ether/DCM gave compound **13** (α:β = 3.5:1) in 80% yield. Treatment of **13** with sodium methoxide (NaOMe) gave rise to building block **4**.

Synthesis of L-fucosamine building block **6** was initiated from known compound **14** (Qin et al., 2018) (Scheme 2). Deacetylation and subsequent dibutyltin oxide-mediated regioselective *p*-methoxybenzylation afforded alcohol **16** in 93% overall yield. Building block **6** was obtained by C4 benzylation and removal of the C3 para-methoxybenzyl (PMB) group in good overall yield.

Assembly of trisaccharide was started from the non-reducing to the reducing end (Scheme 3). The union of thioglycoside donor **5** (David et al., 1989; Wang et al., 2013; Li et al., 2014) and acceptor **6** in the presence of trimethylsilyl trifluoromethanesulfonate (TMSOTf) and *N*-iodosuccinimide (NIS) at 0°C afforded disaccharide **18** in good yield and stereoselectivity. After removal of levulinoyl group (Lev), β-glucosyl derivative was transformed to β-mannosyl derivative **19** by azide displacement of triflate in good overall yield. The anomeric allyl group was removed with palladium(II) chloride (PdCl_2_), followed by introduction of trifluoroacetimidate to give *Yu* donor **3**. TMSOTf-catalyzed glycosylation of **4** with *Yu* donor **3** in a blended-solvents system including DCM, diethyl ether, and thiophene afforded trisaccharide **20** in 82% yield and good stereoselectivity. Reduction of the azido groups with propane-1,3-dithiol and subsequent acetylation gave trisaccharide **21**. 4,6-*O*-benzylidene was removed to afford diol compound, which was oxidized and benzylated at C6 position in good overall yield. Acetylation of alcohol **22** afforded compound **2**, which was transformed to target trisaccharide **1** through global deprotection in 97% yield. The NMR data (^1^H and ^13^C NMR spectra) of trisaccharide **1** are in agreement with those of the isolated polysaccharide (Jones, 2005) (for details, see Supplementary Material, Table S1). The slight spectral differences are most evident toward the reducing end and probably arise due to the installation of aminopropyl linker in the synthetic trisaccharide.

### Preparation and Immunogenicity of Glycoconjugate of Trisaccharide 1

**Scheme 4:**
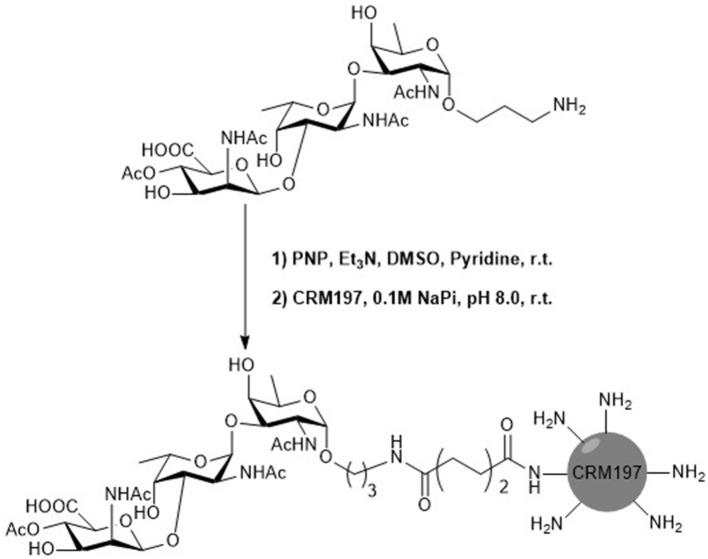
Synthesis of trisaccharide **1**-CRM197.

In order to test the immunogenicity of synthetic hapten, the trisaccharide **1** was covalently linked with immunogenic carrier protein CRM197 to obtain CRM197–trisaccharide **1** glycoconjugate. CRM197 is a Food and Drug Administration (FDA)-approved constitute in marketed carbohydrate conjugate vaccines (Broecker et al., 2011). The glycoconjugate was generated by the coupling of the spacer bis(4-nitrophenyl) adipate with amine group of trisaccharide **1** and lysine amino groups of CRM197 (Scheme 4). The glycoconjugate was confirmed by SDS-PAGE. And the glycan loading on CRM197 was analyzed by MALDI-TOF-MS (Figure S1). The mass spectrum showed the mass peak of glycoconjugate about 63.6 kDa, which indicated around six trisaccharides loaded onto CRM197.

**Figure 2:**
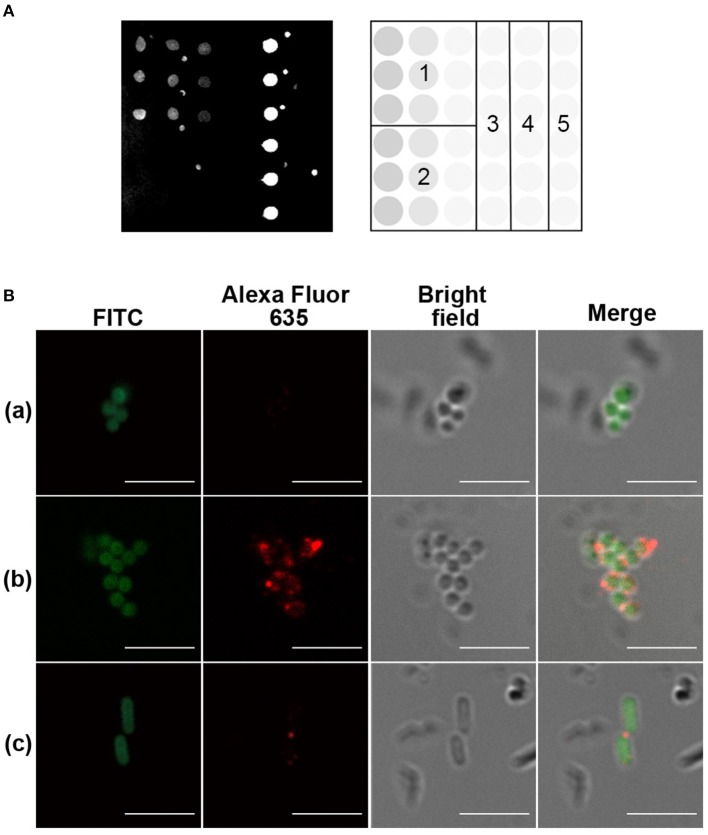
Evaluation of the glycoconjugate in mice. **(A)** Trisaccharide **1**–CRM197 immunized mouse serum, assessed by glycan microarray. The microarray printing pattern is shown to the right of the scan. 1: trisaccharide **1 (**1, 0.5, 0.1 mM), 2: mannose **(**1, 0.5, 0.1 mM), 3: buffer, 4: CRM197 (1 μM), 5: mannose–spacer–bovine serum albumin (BSA) (1 μM). Buffer was 50 mM sodium phosphate, pH 8.5. **(B)** Immunofluorescence of inactivated, fluorescein isothiocyanate (FITC)-labeled bacteria by 1:50 diluted pre- or post-immune serum of glycoconjugate immunized mouse. Scale bar, 5 μm. (a) *Staphylococcus aureus* serotype 8 (ATCC 49525), pre-immune serum. (b) *S. aureus* serotype 8 (ATCC 49525), post-immune serum. (c) *Escherichia coli* (BL21), post-immune serum. Image is representative of *n* ≥ 3 independent experiments with similar results.

In the immunization experiment, each mouse of the immunized group was immunized with one priming dose of CRM197–trisaccharide **1** glycoconjugate containing 4 μg glycan content in complete Freund's adjuvant and one boosting dose of the conjugate in incomplete Freund's adjuvant. Mice of the sham immunized group received the same volume of PBS in the formulation. The serum antibody titers were monitored by glycan microarray. The glycan array results revealed that the immunized mice had increased IgG response specifically against the trisaccharide antigen comparing to pre-immunized serum levels and the sham immunized mice (Figure S2A). Mouse 6 with the highest IgG antibody level was boosted once more and sacrificed to collect the spleen for mAb development (Figure S2B). The serum of endpoint was further analyzed and showed robust IgG response against CRM197 and glycan hapten (Figure 2). No antibody against the spacer constructed in the glycoconjugate was detected. Several glycans, including *E. coli* O55:B5 lipopolysaccharide (LPS), *Plesiomonas shigelloides* (*P. shigelloides*) serotype 51 O-antigen trisaccharide, α-1-6-glucose trisaccharide, and mannose, were selected as control on the glycan microarrays (Figure S3). α-1-6-Glucose trisaccharide is known as an immunodeterminant of *Helicobacter pylori* LPS core oligosaccharide. *P. shigelloides* serotype 51 O-antigen trisaccharide, a zwitterionic oligosaccharide comprising diamino-D-glucuronic acid, L-fucosamine, and D-quinovosamine, served to elucidate the structural specificity of antibodies against trisaccharide **1**. No cross reactivity was detected against control glycans, confirming that immunological specificity of trisaccharide **1** relied on its monosaccharide types, substituents, and glycosidic bonds.

### Immunological Evaluation on Inactivated Bacteria

The mAb was prepared by hybridoma development and subcloning. Several selected hybridoma supernatants containing secreted antibodies were evaluated for binding to *S. aureus* Type 8 trisaccharide **1** by glycan microarray analysis. One selected hybridoma clone cell was intraperitoneally injected to Balb/c mice to collect ascitic fluid for large-scale preparation of mAbs. The ascitic fluid was further purified by sequential precipitation with caprylic acid and ammonium sulfate (Perosa et al., 1990) to obtain the purified mAbs with a concentration of 0.75 mg·mL^−1^ (Figure S3).

**Figure 3:**
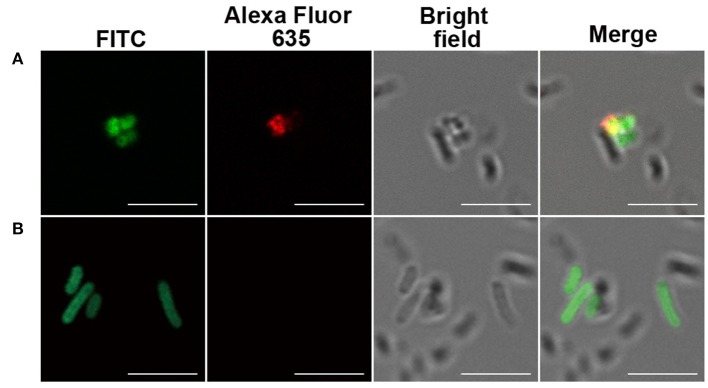
Immunofluorescence of inactivated, fluorescein isothiocyanate (FITC)-labeled bacteria by monoclonal antibodies (mAbs). **(A)** *Staphylococcus aureus* serotype 8 (ATCC 49525) and **(B)** *Escherichia coli* (BL21). Scale bar, 5 μm. Image is representative of *n* ≥ 3 independent experiments with similar results.

Inspired with the glycan microarray results, the bacteria recognition of the serum antibodies and purified mAbs was analyzed by immunofluorescence and imaged with confocal laser scanning microscopy (CLSM). A widely distributed bacteria *E. coli* BL21 strain was used as control. The bacteria were observed by localized green fluorescence on the bacterial surface after direct FITC labeling. The binding of serum IgG antibodies and purified mAbs with the bacteria was detected by the co-localization of red fluorescence after incubation of a goat anti-mouse IgG–Alexa Fluor 635. As shown in Figure 2B and Figure 3, both serum antibodies and purified mAbs bound significantly to the bacterial surface of *S. aureus*, indicating the good recognition of antibodies. Serum antibodies showed very weak binding with *E. coli* BL21, which was probably due to the weak immune response against *E. coli*, but no purified mAbs bound to the control bacteria, implying the specificity of mAbs.

## Conclusion

Based on *S. aureus* CP8 trisaccharide repeating unit, trisaccharide **1** equipped with an anomeric linker was synthesized. A β-glycosylation-epimerization strategy served well to introduce the β-mannosyl into disaccharide intermediate. Two 1,2-*cis*-α-glycosidic linkages were stereoselectively formed that relied on a non-participating C2 azide group and solvent effects of diethyl ether and thiophene. Here, we have further shown the immunogenicity of the synthetic *S. aureus* CP8 trisaccharide **1**. After conjugation with CRM197, the synthetic trisaccharide **1** elicited IgG antibodies in mice. The prepared mAbs have great potential to develop novel therapeutic or preventive approaches with the high specificity with *S. aureus*. The synthetic trisaccharide **1** can be the suitable candidate antigen for the development of carbohydrate-based vaccine against *S. aureus*.

## Data Availability Statement

All datasets generated for this study are included in the article/Supplementary Material.

## Ethics Statement

The animal study was reviewed and approved by Jiangnan University of Technology Animal Care and Use Committee.

## Author Contributions

MZ performed animal studies, generation of mAbs, glycan microarray screening, and bacterial recognition with the help of LL, HX, ZZ, and BM. CQ synthesized *S. aureus* CP8 trisaccharide **1**. JY and JH designed and initiated this study. CQ, JY, and JH wrote the manuscript with input from all authors.

## Conflict of Interest

The authors declare that the research was conducted in the absence of any commercial or financial relationships that could be construed as a potential conflict of interest.
