# Supplementary material for: Conjugation of Synthetic Trisaccharide of Staphylococcus aureus Type 8 Capsular Polysaccharide Elicits Antibodies Recognizing Intact Bacterium
Source: Front Chem. 2020 Apr 28;8:258. doi: 10.3389/fchem.2020.00258 (PMC7199654; doi:10.3389/fchem.2020.00258)

Supplementary Material

**Conjugation of synthetic trisaccharide of Staphylococcus aureus type 8 capsular polysaccharide elicits antibodies recognizing intact bacterium**

Ming Zhao^1#^, Chunjun Qin^1#^, Lingxin Li^1^, Haotian Xie^2^, Beining Ma^2^, Ziru Zhou^2^, Jian Yin^1*^, Jing Hu^2*^

^1^Key Laboratory of Carbohydrate Chemistry and Biotechnology Ministry of Education, School of Biotechnology, Jiangnan University, Wuxi 214122, China.

^2^Wuxi School of Medicine, Jiangnan University, Wuxi 214122, China

# These authors contributed equally.

* Corresponding Author: [jianyin@jiangnan.edu.cn](mailto:jianyin@jiangnan.edu.cn), hujing@jiangnan.edu.cn

# Synthesis of D-fucosamine 4

Phenyl 2-azido-3-*O*-acetyl-2-deoxy-1-seleno-α-D-glucopyranoside (**S1**)





A suspension of **7** (6 g, 12.6 mmol) in 80% HOAc (94 mL) was heated to 55 °C with stirring until starting material was consumed. After evaporation to remove solvent, the residue was purified by silica gel column chromatography (petroleum ether : ethyl acetate 2 : 1 *v*/*v*) to afford product **S1** (4.9 g, 12.6 mmol, quant.). ^1^H NMR (400 MHz, CDCl_3_) δ = 7.26 (s, 5H, Ar-H), 5.89 (d, *J* = 5.4 Hz, 1H, 1-H), 5.11 (dd, *J* = 10.2, 9.2 Hz, 1H, 3-H), 4.16 (dt, *J* = 9.8, 3.8 Hz, 1H, 5-H), 3.97 (dd, *J* = 10.2, 5.4 Hz, 1H, 2-H), 3.80 (ddd, *J* = 5.5, 3.8, 1.8 Hz, 2H, 6-CH_2_), 3.71 (td, *J* = 9.6, 5.5 Hz, 1H, 4-H), 3.01 (d, *J* = 5.7 Hz, 1H, 4-OH), 2.21 (s, 3H, CH_3_), 1.76 (t, *J* = 6.4 Hz, 1H, 6-OH); ^13^C NMR (100 MHz, CDCl_3_) δ = 172.1, 135.0, 129.3, 128.3, 83.7, 77.2, 76.6, 74.3, 69.8, 62.2, 61.7, 21.0.

Phenyl 2-azido-3-*O*-acetyl-2-deoxy-6-*O*-(*p*-toluenesulfonyl)-1-seleno-α-D-glucopyranoside (**8**)





To a solution of **S1** (4.9 g, 12.6 mmol) in anhydrous pyridine (90 mL), *p*-toluene-sulfonyl chloride (4.1 g, 21.4 mmol) was added under argon. The reaction mixture was stirred at room temperature overnight. The mixture was diluted with ethyl acetate (100 mL), washed with 1M aq. HCl (2 × 150 mL), satd. aq. NaHCO_3_ (3 × 150 mL) and water (3 × 150 mL), then dried (Na_2_SO_4_), filtered and concentrated. The residue was purified by silica gel column chromatography (petroleum ether : ethyl acetate 4 : 1 *v*/*v*) to give product **8** (5.9 g, 10.9 mmol, 87%). ^1^H NMR (400 MHz, CDCl_3_) δ = 7.26 (s, 9H, Ar-H), 5.81 (d, *J* = 5.4 Hz, 1H, 1-H), 5.06 (t, *J* = 9.7 Hz, 1H, 3-H), 4.35 (dd, *J* = 11.2, 4.1 Hz, 1H, 6-H_a_), 4.28 (ddd, *J* = 9.9, 4.1, 1.8 Hz, 1H, 5-H), 4.10 (dd, *J* = 11.0, 1.9 Hz, 1H, 6-H_b_), 3.93 (dd, *J* = 10.2, 5.4 Hz, 1H, 2-H), 3.69 (td, *J* = 9.5, 5.5 Hz, 1H, 4-H), 3.01 (d, *J* = 5.5 Hz, 1H, 4-OH), 2.44 (s, 3H, CH_3_), 2.20 (s, 3H, CH_3_).

Phenyl 2-azido-3-*O*-acetyl-2,6-dideoxy-6-iodo-1-seleno-α-D-glucopyranoside (**S2**)





Tosylate compound **8** (5.9 g, 10.9 mmol) was refluxed for 3 h in butanone (134 mL) together with NaI (8.1 g, 53.7 mmol) and AcOH (0.8 mL) under argon. After cooling to room temperature, DCM (100 mL) was added and the mixture was washed with aq. 1M Na_2_S_2_O_3_ solution (3 × 200 mL) and water (2 × 150 mL). The organic phase was dried (Na_2_SO_4_), filtered, and concentrated. The residue was purified by silica gel column chromatography (petroleum ether : ethyl acetate 7 : 1 *v*/*v*) to give product **S2** (4.9 g, 9.8 mmol, 91%). ^1^H NMR (400 MHz, CDCl_3_) δ = 7.26 (s, 5H, Ar-H), 5.90 (d, *J* = 5.3 Hz, 1H, 1-H), 5.09 (dd, *J* = 10.2, 9.1 Hz, 1H, 3-H), 3.99 (dd, *J* = 10.2, 5.3 Hz, 1H, 2-H), 3.84 (ddd, *J* = 9.3, 4.8, 2.9 Hz, 1H, 5-H), 3.57 (t, *J* = 9.2 Hz, 1H, 4-H), 3.48 (dd, *J* = 11.0, 4.8 Hz, 1H, 6-H_a_), 3.41 (dd, *J* = 11.0, 2.9 Hz, 1H, 6-H_b_), 2.22 (s, 3H, CH_3_); ^13^C NMR (100 MHz, CDCl_3_) δ = 172.3, 134.7, 129.3, 128.2, 127.8, 84.0, 77.2, 76.3, 73.6, 72.5, 62.2, 29.7, 20.9, 6.4.

Phenyl 2-azido-3-*O*-acetyl-2-deoxy-1-seleno-α-D-quinovopyranoside (**9**)





To a solution of compound **S2** (4.8 g, 9.7 mmol) in anhydrous DMF (108 mL) under argon, sodium cyanoborohydride (4.8 g, 77.9 mmol) was added and the reaction mixture stirred at 95 °C for 3 h. The mixture was poured into water (80 mL) and extracted with DCM (2 × 200mL). The organic phase was dried over Na_2_SO_4_ and concentrated. The residue was purified by silica gel column chromatography (petroleum ether : ethyl acetate 7 : 1 *v*/*v*) to give product **9** (1.4 g, 3.9 mmol, 40%). ^1^H NMR (400 MHz, CDCl_3_) δ = 7.86 - 6.91 (m, 5H, Ar-H), 5.82 (d, *J* = 5.3 Hz, 1H, 1-H), 5.04 (dd, *J* = 10.2, 9.2 Hz, 1H, 3-H), 4.15 (dq, *J* = 9.6, 6.2 Hz, 1H, 4-H), 3.97 (dd, *J* = 10.2, 5.4 Hz, 1H, 2-H), 3.33 (td, *J* = 9.4, 5.9 Hz, 1H, 5-H), 2.66 (d, *J* = 6.0 Hz, 1H, 4-OH), 2.21 (s, 3H, CH_3_), 1.28 (d, *J* = 6.2 Hz, 3H, CH_3_); ^13^C NMR (100 MHz, CDCl_3_) δ = 172.3, 134.7, 129.2, 128.1, 84.0, 78.5, 75.3, 75.0, 71.0, 62.5, 21.0, 17.2.

Phenyl 2-azido-3-*O*-acetyl-4-*O*-trifluoromethanesulfonyl-2-deoxy-1-seleno-α-D-quinovopyranoside (**S3**)





To a solution of **9** (1.1 g, 2.8 mmol) in anhydrous DCM (20 mL), pyridine (2 mL) was added at -20 °C under argon. Tf_2_O (1 mL, 5.9 mmol) was added dropwise, and the mixture was stirred while allowing to warm from -20 °C to -10 °C over 2 h. The resulting mixture was diluted with DCM (20 mL) washed with 1M aq. HCl (2 × 50 mL), satd. aq. NaHCO_3_ (2 × 50 mL) and water (2 × 60 mL). The organic phase was dried over Na_2_SO_4_ and concentrated under vacuum at 30 °C. The residue was purified by silica gel column chromatography (petroleum ether : ethyl acetate 20 : 1 *v*/*v*) to afford product **S3** (1.1 g, 2.1 mmol, 74%). ^1^H NMR (400 MHz, CDCl_3_) δ = 7.90 - 6.90 (m, 5H, Ar-H), 5.86 (d, *J* = 5.5 Hz, 1H, 1-H), 5.45 (t, *J* = 9.7 Hz, 1H, 3-H), 4.59 (t, *J* = 9.5 Hz, 1H, 4-H), 4.55 - 4.43 (m, 1H, 2-H), 4.00 (dd, *J* = 10.2, 5.5 Hz, 1H, 5-H), 2.19 (s, 3H, CH_3_), 1.31 (d, *J* = 6.1 Hz, 3H, CH_3_); ^13^C NMR (100 MHz, CDCl_3_) δ = 169.3, 134.8, 129.4, 128.4, 127.3, 84.6, 83.2, 78.9, 75.0, 70.9, 67.7, 63.3, 20.6, 17.0.

Phenyl 2-azido-3-*O*-acetyl-2-deoxy-1-seleno-α-D-fucopyranoside (**10**)





KNO_2_ (0.9 g, 10.0 mmol) was added to a solution of triflate **S3** (1 g, 2.0 mmol) in anhydrous DMF (20 mL) under argon. After stirring at 50 °C for 1 h, the mixture was diluted with DCM (10 mL) and washed with brine (2 × 40 mL). The organic phase was dried over Na_2_SO_4_ and concentrated under vacuum. Purification of the residue by silica gel column chromatography (petroleum ether : ethyl acetate 5 : 1 *v*/*v*) afforded the inversion product **10** (514 mg, 1.4 mmol, 69%). ^1^H NMR (400 MHz, CDCl_3_) δ = 7.26 (s, 5H, Ar-H), 5.93 (d, *J* = 5.5 Hz, 1H, 1-H), 5.06 (dd, *J* = 10.8, 3.0 Hz, 1H, 3-H), 4.43 (q, *J* = 6.6 Hz, 1H, 5-H), 4.31 (dd, *J* = 10.7, 5.4 Hz, 1H, 2-H), 3.99 (s, 1H, 4-H), 2.19 (s, 3H, CH_3_), 1.22 (d, *J* = 6.6 Hz, 3H, CH_3_).

Phenyl 2-azido-3-*O*-acetyl-4-*O*-benzyl-2-deoxy-1-seleno-α-D-fucopyranoside (**11**)





Compound **10** (450 mg, 1.2 mmol) was dissolved in anhydrous DCM (12 mL) under argon and at 0 °C, BnBr (1.4 mL, 12.2 mmol) and Ag_2_O (855 mg, 3.6 mmol) were added. The reaction mixture was stirred at 0 °C for 24 h. After filtration through celite and concentration, the residue was purified by silica gel column chromatography (petroleum ether : ethyl acetate 20:1 *v*/*v*) to afford product **11** (249 mg, 0.54 mmol, 45%). ^1^H NMR (400 MHz, CDCl_3_) δ = 7.68 - 7.21 (m, 10H, Ar-H), 5.91 (d, *J* = 5.3 Hz, 1H, 1-H), 5.46 (dd, *J* = 3.3, 1.2 Hz, 1H, 4-H), 4.75 (d, *J* = 10.7 Hz, 1H, PhCH_2_), 4.53 (d, *J* = 10.7 Hz, 1H, PhCH_2_), 4.48 - 4.37 (m, 1H, 5-H), 4.12 (dd, *J* = 10.3, 5.4 Hz, 1H, 2-H), 3.78 (dd, *J* = 10.4, 3.3 Hz, 1H, 3-H), 2.15 (s, 3H, CH_3_), 1.12 (d, *J* = 6.5 Hz, 3H, CH_3_); ^13^C NMR (100 MHz, CDCl_3_) δ = 136.9, 134.5, 129.1, 128.5, 128.3, 128.1, 127.9, 85.1, 77.3, 71.7, 68.9, 67.8, 60.4, 20.8, 16.1.

*N*-Benzyl-*N*-benzyloxycarbonyl-3-aminopropyl 2-azido-3-*O*-acetyl-4-*O*-benzyl-2-deoxy-D-fucopyranoside (**13**)





A solution of **11** (160 mg, 0.348 mmol) in 1:1 *v*/*v* THF/water (1 mL) was treated with *N*-bromosuccinimide (150 mg, 0.843 mmol). After complete conversion of the starting material, the solution was diluted with DCM (5 mL) and washed with 1:1 *v*/*v* 10% (*w*/*v*) Na_2_S_2_O_3_/1M NaHCO_3_ (3 × 5 mL), filtered and concentrated. The residue was purified by silica gel column chromatography (petroleum ether : ethyl acetate 4:1 *v*/*v*) to afford 1-OH product (108 mg, 0.336 mmol, 97%).

To a solution of above 1-OH compound (108 mg, 0.336 mmol) in anhydrous DCM (4 mL), trichloroacetonitrile (0.3 mL, 2.992 mmol) and DBU (5 μL, 0.033 mmol) were added under argon. The mixture was stirred at room temperature for 4 h, when TLC showed complete conversion. The solvent was evaporated, and the residue was purified by silica gel column chromatography (petroleum ether : ethyl acetate 10:1 *v*/*v*, containing 0.5% Et_3_N) to give imidate **12** (149 mg, 0.320 mmol, 95%)

Trichloroacetimidate donor **12** (149 mg, 0.320 mmol), *N*-Bn-*N*-Cbz-3-aminopropan-1-ol (115 mg, 0.384 mmol) and thiophene (0.3 mL, 3.747 mmol) were dissolved in 3:1 (*v*/*v*) anhydrous Et_2_O/ anhydrous DCM (8 mL) under argon. This solution was treated with flame-dried molecular sieves (AW-300) and was stirred for 30 min. The mixture was cooled to -30 °C and TMSOTf (70 μL, 0.387 mmol) was slowly added. The mixture was stirred under -30 °C for 2 h. The mixture was neutralized with Et_3_N (0.1 mL) under -30 °C and filtered through a pad of celite. The filtrate was washed with satd. aq. NaHCO_3_ (2 × 6 mL), dried over Na_2_SO_4_ and concentrated. The residue was purified by silica gel column chromatography (petroleum ether : ethyl acetate 6 : 1 *v*/*v*) to give coupled product (80%, α:β = 3.5:1) including target α-product **13** (120 mg, 0.199 mmol, 62%). ^1^H NMR (400 MHz, CDCl_3_) δ = 7.41-7.12 (m, 15H, 3Ph), 5.26 (s, 1H, 3-H), 5.22-5.13 (m, 2H, Bn-CH_2_), 4.84 (s, 1H, 1-H), 4.69 (d, *J* =11.3 Hz, 1H, Bn-CH_2_), 4.63-4.39 (m, 3H, Bn-CH_2_), 3.90 (m, 1H, 5-H), 3.76 (m, 3H, 2-H, 4-H, linker-1H), 3.36 (m, 3H, linker-3H), 2.09 (s, 3H, CH_3_CO), 1.87 (m, 2H, linker-2H), 1.14 (s, 3H, 6-CH_3_); ^13^C NMR (100 MHz, CDCl_3_) δ = 170.3, 137.7, 128.6, 128.5, 128.4, 128.2, 127.99, 127.96, 127.90, 127.4, 98.2, 77.2, 75.7, 71.5, 67.3, 66.3, 65.9, 57.7, 51.0, 20.9, 16.4.

*N*-Benzyl-*N*-benzyloxycarbonyl-3-aminopropyl 2-azido-4-*O*-benzyl-2-deoxy-D-fucopyranoside (**4**)





To a solution of **13** (61 mg, 0.10 mmol) in MeOH (2 mL), NaOMe (3 mg, 0.06 mmol) was added and the solution was stirred at room temperature for 2 h. The reaction solution was neutralized with Amberlite IR 120 (H^+^) ion exchange resin and filtered through a cotton plug. The crude mixture was purified by silica gel column chromatography (petroleum ether : ethyl acetate 6 : 1 *v*/*v*) to give product **4** (56 mg, 0.10 mmol, quant.). ^1^H NMR (400 MHz, CDCl_3_) δ = 7.46-7.11 (m, 15H, 3Ph), 5.18 (m, 2H, Bn-2H), 4.78 (m, 2H, Bn-1H, 1-H), 4.65 (d, *J* = 11.5 Hz, 1H, Bn-1H), 4.60-4.39 (m, 2H, Bn-2H), 3.91 (m, 2H, 3-H, 5-H), 3.59 (m, 2H, linker-2H), 3.35 (m, 4H, 2-H, 4-H, linker-2H), 2.05 (d, *J* = 9.0 Hz, 1H, 3-OH), 1.82 (m, 2H, linker-2H), 1.23 (d, *J* = 6.4 Hz, 3H, 6-CH_3_).

# Synthesis of L-fucosamine 6

Allyl 2-azido-2-deoxy-β-L-fucopyranoside (**15**)





To a solution of allyl 3,4-di-*O*-acetyl-2-azido-2-deoxy-β-L-fucopyranoside (**14**) (447 mg, 1.43 mmol) in MeOH (15 mL), NaOMe (39 mg, 0.72 mmol) was added and the solution was stirred at room temperature for 4 h. The reaction solution was neutralized with Amberlite IR 120 (H^+^) ion exchange resin and filtered through a cotton plug. Evaporation under reduced pressure gave product **15** as white solid (327 mg, 1.43 mmol, quant.). [α]_D_^20^ = - 27.6º (c = 1.00, CHCl_3_); IR ν_max_ (film) 3312, 2865, 2111, 1348, 1279, 1162, 1071, 999, 925, 755 cm^-1^; ^1^H NMR (400 MHz, CDCl_3_) δ = 6.04-5.85 (m, 1H, CH=C), 5.34 (dq, *J* = 17.3, 1.6 Hz, 1H, C=CH_a_), 5.23 (dd, *J* = 10.5, 1.6 Hz, 1H, C=CH_b_), 4.41 (ddt, *J* = 12.9, 5.3, 1.5 Hz, 1H, OCH_a_), 4.30 (d, *J* = 7.9 Hz, 1H, 1-H), 4.14 (ddt, *J* = 12.8, 6.1, 1.4 Hz, 1H, OCH_b_), 3.70 (d, *J* = 3.2 Hz, 1H, 4-H), 3.63-3.53 (m, 1H, 5-H), 3.53 (dd, *J* = 10.1, 7.8 Hz, 1H, 2-H), 3.45 (dd, *J* = 10.1, 3.3 Hz, 1H, 3-H), 3.04 (s, 1H, OH), 2.68 (s, 1H, OH), 1.35 (d, *J* = 6.5 Hz, 3H, 6-CH_3_); ^13^C NMR (100 MHz, CDCl_3_) δ = 133.5, 117.8, 101.1, 72.6, 70.9, 70.6, 70.3, 64.0, 16.3; HR-ESI-MS (m/z): calcd for C_9_H_15_N_3_O_4_Na^+^ (M + Na^+^): 252.0960, found: 252.0954.

Allyl 2-azido-3-*O*-4-methoxybenzyl-2-deoxy-β-L-fucopyranoside (**16**)





The diol **15** (0.40 g, 1.74 mmol) was co-evaporated with anhydrous toluene and dried under high vacuum for 3 h. Then, anhydrous toluene (18 mL) was added under argon, followed by Bu_2_SnO (1.4 g, 2.8 mmol) and 4 Å molecular sieves (flame dried). The reaction was stirred for 4 h under reflux. The reaction was cooled to room temperature, PMBCl (0.55 mL, 2.2 mmol) and TBAB (0.65 g, 2.0 mmol) were added and stirred for 3 h under reflux. The reaction was filtered and the solvent was evaporated. The residue was purified by silica gel column chromatography (petroleum ether : ethyl acetate 5 : 1 *v*/*v*) to afford **16** (0.56 g, 1.60 mmol, 92%). ^1^H NMR (400 MHz, CDCl_3_) δ = 7.26 (s, 4H, Ar-H), 6.05 - 5.86 (m, 1H, allyl-1H), 5.38 - 5.28 (m, 1H, allyl-1H), 5.21 (dd, *J* = 10.4, 1.5 Hz, 1H, allyl-1H), 4.64 (s, 2H, PhCH_2_), 4.43 - 4.34 (m, 1H, allyl-1H), 4.22 (d, *J* = 8.1 Hz, 1H, 1-H), 4.16 - 4.05 (m, 1H, allyl-1H), 3.81 (s, 3H, CH_3_), 3.73 - 3.66 (m, 1H, 4-H), 3.59 (dd, *J* = 10.0, 8.1 Hz, 1H, 2-H), 3.47 (q, *J* = 6.5 Hz, 1H, 5-H), 3.30 (dd, *J* = 10.0, 3.3 Hz, 1H, 3-H), 2.34 - 2.25 (m, 1H, 4-OH).

Allyl 2-azido-4-*O*-benzyl-3-*O*-4-methoxybenzyl-2-deoxy-β-L-fucopyranoside (**17**)





To a solution of **16** (3.6 g, 10.3 mmol) in anhydrous DMF (60 mL) were added NaH (530 mg, 20.7 mmol) at 0 °C for 30 min. Then benzyl bromide (2 mL, 15.5 mmol) was added to the reaction mixture and stirred at room temperature for 3 h. Upon reaction completion, the mixture was diluted with DCM (30 mL), added water (100 mL), and extracted with DCM (3 × 100 mL). Combined organic layer was washed with brine (2 × 200mL), dried over Na_2_SO_4_ and concentrated. The residue was purified by silica gel column chromatography (petroleum ether : ethyl acetate 10 : 1 *v*/*v*) to afford **17** (3.2 g, 7.3 mmol, 71%). ^1^H NMR (400 MHz, CDCl_3_) δ = 7.59 - 6.74 (m, 9H, Ar-H), 5.93 (dddd, *J* = 16.9, 10.9, 6.1, 5.0 Hz, 1H, allyl-1H), 5.31 (dq, *J* = 17.2, 1.7 Hz, 1H, allyl-1H), 5.22 - 5.17 (m, 1H, allyl-1H), 4.93 (d, *J* = 11.7 Hz, 1H, PhCH_2_), 4.70 - 4.52 (m, 3H, PhCH_2_), 4.38 (ddt, *J* = 12.9, 5.1, 1.6 Hz, 1H, allyl-1H), 4.21 (d, *J* = 8.0 Hz, 1H, 1-H), 4.14 - 4.04 (m, 1H, allyl-1H), 3.88 - 3.76 (m, 4H, 2-H, CH_3_), 3.50 (dd, *J* = 2.9, 1.0 Hz, 1H, 4-H), 3.45 - 3.36 (m, 1H, 5-H), 3.29 (dd, *J* = 10.4, 2.8 Hz, 1H, 3-H), 1.19 (d, *J* = 6.4 Hz, 3H, CH_3_); ^13^C NMR (100 MHz, CDCl_3_) δ = 138.3, 133.8, 129.8, 129.5, 128.4, 128.2, 127.7, 117.4, 113.9, 100.9, 80.8, 74.9, 74.6, 72.3, 70.6, 69.9, 63.0, 55.3, 16.9.

Allyl 2-azido-4-*O*-benzyl-2-deoxy-β-L-fucopyranoside (**6**)





To a solution of **17** (3.2 g, 7.3 mmol) in DCM (300 mL) were added DDQ (2.5 g, 11.0 mmol) and water (18 mL). The reaction mixture was stirred at room temperature for 4 h. Upon reaction completion, the mixture was diluted with DCM (100 mL), washed with 5% (*w*/*v*) Na_2_S_2_O_3_ (2 × 400 mL), dried over Na_2_SO_4_ and concentrated. The residue was purified by silica gel column chromatography (petroleum ether : ethyl acetate 10 : 1 *v*/*v*) to afford **6** (2.1 g, 6.6 mmol, 90%). ^1^H NMR (400 MHz, CDCl_3_) δ = 7.46 - 7.18 (m, 5H, Ph), 5.94 (dddd, *J* = 16.9, 10.9, 6.1, 5.1 Hz, 1H, allyl-1H), 5.33 (dd, *J* = 17.2, 1.7 Hz, 1H, allyl-1H), 5.21 (dd, *J* = 10.4, 1.5 Hz, 1H, allyl-1H), 4.82 (d, *J* = 11.6 Hz, 1H, PhCH_2_), 4.72 (d, *J* = 11.6 Hz, 1H, PhCH_2_), 4.41 (ddt, *J* = 12.9, 5.0, 1.5 Hz, 1H, allyl-1H), 4.26 (d, *J* = 7.9 Hz, 1H, 1-H), 4.18 - 4.06 (m, 1H, allyl-1H), 3.64 - 3.48 (m, 3H, 3-H, 4-H, 5-H), 3.45 (ddd, *J* = 10.2, 7.7, 3.4 Hz, 1H, 2-H), 2.22 (d, *J* = 7.7 Hz, 1H, 3-OH), 1.32 (d, *J* = 6.5 Hz, 3H, CH_3_); ^13^C NMR (100 MHz, CDCl_3_) δ = 137.9, 133.6, 128.6, 128.2, 128.1, 117.5, 101.0, 78.5, 76.0, 73.0, 70.9, 70.1, 64.6, 16.9.

# Synthesis of trisaccharide 21

Allyl 2-azido-4-*O*-benzyl-3-*O*-(4,6-*O*-benzylidene-3-*O*-benzyl-2-*O*-levulinoyl-β-D-glucopyranosyl)-2-deoxy-β-L-fucopyranoside (**18**)





To a solution of **5** (180 mg, 0.32 mmol) and **6** (51 mg, 0.16 mmol) in anhydrous DCM (7 mL) were added flame-dried molecular sieve (AW-300) and the reaction mixture was stirred at room temperature under argon for 30 min. The mixture was cooled to 0 °C and *N*-iodosuccinimide (86 mg, 0.38 mmol) and TMSOTf (29 μL, 0.16 mmol) were added. After stirring at the same temperature for 4 h, Et_3_N (0.5 mL) was added to quench the reaction. The mixture was filtered through a pad of celite and washed with DCM. The organic layer was successively washed with 5% (*w*/*v*) Na_2_S_2_O_3_ (3 × 5 mL), satd. aq. NaHCO_3_ (2 × 5 mL), then dried (Na_2_SO_4_) and evaporated to dryness. The residue was purified by silica gel column chromatography (petroleum ether : ethyl acetate 4 : 1 *v*/*v*) to give disaccharide **18** (107 mg, 0.14 mmol, 88%, β only). ^1^H NMR (400 MHz, CDCl_3_) δ = 7.55-7.25 (m, 15H, 3Ph), 5.94 (m, 1H, allyl-1H), 5.60 (s, 1H, PhCH), 5.32 (m, 1H, allyl-1H), 5.21-5.16 (m, 1H, allyl-1H), 5.10 (dd, *J* = 8.5, 7.4 Hz, 1H, 2’-H), 4.93-4.83 (m, 2H, Bn-2H), 4.80 (d, *J* = 7.7 Hz, 1H, 1’-H), 4.70 (d, *J* = 11.7 Hz, 1H, Bn-1H), 4.52 (d, *J* = 11.3 Hz, 1H, Bn-1H), 4.46 (dd, *J* = 10.4, 5.2 Hz, 1H, 6’-CH_a_), 4.39 (m, 1H, allyl-1H), 4.22 (d, *J* = 8.0 Hz, 1H, 1-H), 4.11 (dd, *J* = 13.0, 6.3 Hz, 1H, allyl-1H), 3.91 (m, 2H, 6’-CH_b_, 4’-H), 3.83-3.68 (m, 2H, 3’-H, 2-H), 3.63 (dd, *J* = 10.3, 2.7 Hz, 1H, 3-H), 3.54 (m, 2H, 4-H, 5’-H), 3.48 (q, *J* = 6.4, 5.7 Hz, 1H, 5-H), 2.69-2.25 (m, 4H, Lev-CH_2_), 2.06 (s, 3H, lev-CH_3_), 1.18 (d, *J* = 6.5 Hz, 3H, 6-CH_3_).

Allyl 2-azido-4-*O*-benzyl-3-*O*-(4,6-*O*-benzylidene-3-*O*-benzyl-β-D-glucopyranosyl)-2-deoxy-β-L-fucopyranoside (**S4**)





To a solution of **18** (0.34 g, 0.450 mmol) in DCM/MeOH (20/1 *v*/*v*) (5 mL) was added hydrazine acetate (51 mg, 0.55 mmol). The reaction mixture was stirred for 5 h at room temperature. Upon reaction completion, the solvent was evaporated under reduced pressure. The residue was purified by silica gel column chromatography (petroleum ether : ethyl acetate 8 : 1 *v*/*v*) to give product **S4** (222 mg, 0.337 mmol, 75%). ^1^H NMR (400 MHz, CDCl_3_) δ = 7.55-7.27 (m, 15H, 3Ph), 5.94 (m, 1H, allyl-1H), 5.60 (s, 1H, PhCH), 5.33 (d, *J* = 16.9 Hz, 1H, allyl-1H), 5.21 (d, *J* = 10.2 Hz, 1H, allyl-1H), 5.00 (m, 2H, Bn-2H), 4.78 (d, *J* = 11.5 Hz, 1H, Bn-1H), 4.75 (d, *J* = 11.4 Hz, 1H, Bn-1H), 4.64 (d, *J* = 5.3 Hz, 1H, 1’-H), 4.40 (m, 2H, allyl-1H, 6’-CH_a_), 4.26 (d, *J* = 7.8 Hz, 1H, 1-H), 4.09 (dd, *J* = 12.9, 6.1 Hz, 1H, allyl-1H), 3.83 (m, 2H, 3’-H, 6’-CH_b_), 3.79-3.63 (m, 4H, 4’-H, 2-H, 3-H, 5’-H), 3.60 (d, *J* = 2.7 Hz, 1H, 4-H), 3.56-3.38 (m, 2H, 2’-H, H-5), 2.37-2.27 (m, 1H, 2’-OH), 1.20 (d, *J* = 6.5 Hz, 3H, 6-CH_3_); ^13^C NMR (100 MHz, CDCl_3_) δ = 138.31, 138.29, 137.2, 133.7, 129.7, 129.1, 128.47, 128.45, 128.3, 128.24, 128.20, 128.0, 127.9, 127.7, 126.0, 117.7, 101.7, 101.3, 100.8, 81.2, 80.4, 78.6, 77.2, 75.5, 74.72, 74.66, 73.2, 70.5, 70.0, 68.7, 66.9, 61.9, 16.8.

Allyl 2-azido-4-*O*-benzyl-3-*O*-(2-azido-4,6-*O*-benzylidene-3-*O*-benzyl-2-deoxy-β-D-mannopyranosyl)-2-deoxy-β-L-fucopyranoside (**19**)





To a solution of **S4** (139 mg, 0.211 mmol) in anhydrous DCM (1.5 mL) was added pyridine (0.3 mL) at -20 °C under argon. Tf_2_O (0.1 mL, 0.585 mmol) was added dropwise, and the mixture was stirred while allowing to warm from -20 °C to 10 °C. Upon reaction completion, the resulting mixture was subsequently diluted with DCM (5 mL) and washed with 1M aq. HCl (10 mL), satd. aq. NaHCO_3_ (2 × 10 mL). The organic phase was dried over Na_2_SO_4_ and concentrated under vacuum at 30 °C. The residue was purified by silica gel column chromatography (petroleum ether : ethyl acetate 5 : 1 *v*/*v*) to afford triflate compound (107 mg, 0.134 mmol, 64%).

Triflate compound (107 mg, 0.134 mmol) was dissolved in anhydrous DMF (2 mL) under argon, NaN_3_ (57 mg, 0.88 mmol) was added, and the resulting mixture was stirred at 60 °C for 3 h. Upon reaction completion, the mixture was diluted with DCM (6 mL), washed with water (2 × 10 mL), dried with Na_2_SO_4_ and concentrated. The residue was purified by silica gel column chromatography (petroleum ether : ethyl acetate 5 : 1 *v*/*v*) to give product **19** (81 mg, 0.120 mmol, 90%). ^1^H NMR (400 MHz, CDCl_3_) δ = 7.51-7.27 (m, 15H, 3Ph), 5.95 (m, 1H, allyl-1H), 5.58 (s, 1H, PhCH), 5.34 (dd, *J* = 17.2, 1.9 Hz, 1H, allyl-1H), 5.22 (dd, *J* = 10.7, 1.7 Hz, 1H, allyl-1H), 4.85 (d, *J* = 12.6 Hz, 1H, Bn-1H), 4.80 (d, *J* = 11.9 Hz, 1H, Bn-1H), 4.74 (d, *J* = 12.5 Hz, 1H, Bn-1H), 4.64 (d, *J* = 11.8 Hz, 1H, Bn-1H), 4.45-4.39 (m, 1H, allyl-1H), 4.38 (d, *J* = 1.2 Hz, 1H, 1’-H), 4.31 (dd, *J* = 10.4, 4.8 Hz, 1H, 6’-CH_a_), 4.26 (d, *J* = 7.5 Hz, 1H, 1-H), 4.17-4.06 (m, 1H, allyl-1H), 4.04 (t, *J* = 9.3 Hz, 1H, 4’-H), 3.91 (t, *J* = 10.1 Hz, 1H, 6’-CH_b_), 3.77 (dd, *J* = 10.6, 7.8 Hz, 1H, 2-H), 3.69 (dd, *J* = 10.2, 3.1 Hz, 1H, 3-H), 3.60-3.51 (m, 2H, 4-H, 3’-H), 3.51-3.39 (m, 2H, 2’-H, 5-H), 3.21 (td, *J* = 9.5, 4.6 Hz, 1H, 5’-H), 1.35 (d, *J* = 6.2 Hz, 3H, 6-CH_3_); ^13^C NMR (100 MHz, CDCl_3_) δ = 138.1, 137.8, 137.2, 133.7, 129.0, 128.53, 128.51, 128.47, 128.45, 128.41, 128.37, 128.3, 128.2, 128.1, 127.98, 127.95, 127.8, 127.7, 126.0, 117.5, 101.5, 101.0, 100.9, 97.0, 78.3, 77.6, 76.3, 75.4, 75.0, 72.9, 70.4, 70.1, 68.3, 67.68, 67.65, 63.3, 62.1, 17.1.

2-Azido-4-*O*-benzyl-3-*O*-(2-azido-4,6-*O*-benzylidene-3-*O*-benzyl-2-deoxy-β-D-mannopyranosyl)-2-deoxy-β-L-fucopyranosyl 1-(*N*-phenyl)-2,2,2-trifluoroacetimidate (**3**)





To a solution of **19** (26 mg, 0.038 mmol) in 20:1 (v/v) AcOH/water (3 mL) were added AcONa (183 mg, 2.244 mmol) and PdCl_2_ (11.4 mg, 0.065 mmol). The reaction mixture was stirred at room temperature for 3 h. Upon reaction completion, the mixture was diluted with ethyl acetate (6 mL) and filtered (celite). The organic phase was washed with satd. aq. NaHCO_3_ (2 × 5 mL), dried over Na_2_SO_4_ and concentrated under vacuum at 30 °C. The residue was purified by silica gel column chromatography (petroleum ether : ethyl acetate 2 : 1 *v*/*v*) to afford hemiacetal compound (20 mg, 0.031 mmol, 82%).

A solution of hemiacetal (20 mg, 0.031 mmol), *N*-phenyl trifluoroacetimidoyl chloride (10 μL, 0.067 mmol) and DBU (11 μL, 0.074 mmol) in anhydrous DCM (2 mL) under argon was stirred at room temperature for 10 h. The reaction mixture was concentrated under vacuum to give a residue, which was purified by silica gel column chromatography (petroleum ether : ethyl acetate 5 : 1 *v*/*v*) to afford **3** (15 mg, 0.018 mmol, 58%).

*N*-Benzyl-*N*-benzyloxycarbonyl-3-aminopropyl 4-*O*-benzyl-3-*O*-(4-*O*-benzyl-3-*O*-[2-azido-4,6-*O*-benzylidene-3-*O*-benzyl-2-deoxy-β-D-mannopyranosyl]-2-azido-2-deoxy-α-L-fucopyranosyl)-2-azido-2-deoxy-α-D-fucopyranoside (**20**)





Trifluoroacetimidate donor **3** (60 mg, 0.074 mmol) and acceptor **4** (30 mg, 0.054 mmol) were dissolved in anhydrous DCM / anhydrous Et_2_O (*v*/*v*, 1/3, 2.5 mL) under argon, thiophene (70 μL, 0.874 mmol) and flame-dried molecular sieves (AW-300) were added. The mixture was stirred at room temperature for 30 min. After cooling to -10 °C, TMSOTf (1.5 μL, 0.008 mmol) was added. The reaction mixture was stirred at -10 °C for 5 h. Et_3_N (0.5 mL) was added to neutralize the reaction, and filtered through a pad of celite. The reaction mixture was washed with satd. aq. NaHCO_3_ (2 × 5 mL), dried over Na_2_SO_4_ and concentrated. The residue was purified by silica gel column chromatography (petroleum ether : ethyl acetate 3 : 1 *v*/*v*) to afford **20** (52 mg, 0.044 mmol, 82%, α only). ^1^H NMR (400 MHz, CDCl_3_) δ = 7.63-7.15 (m, 30H, 6Ph), 5.58 (s, 1H, PhCH), 5.21 (d, *J* = 4.2 Hz, 1H, 1'-H), 5.19-5.12 (m, 2H, Bn-2H), 4.88 (m, 2H, 1-H, Bn-1H), 4.79 (d, *J* = 11.2 Hz, 1H, Bn-1H), 4.76-4.60 (m, 4H, Bn-4H), 4.56 (m, 3H, 1''-H, NBn-2H), 4.17 (m, 2H, 3'-H, 6''-CH_a_), 4.06 (m, 2H, 4''-H, 3-H), 3.91 (m, 2H, 5-H, 5'-H), 3.83 (m, 2H, 2-H, 6''-CH_b_), 3.73 (dd, *J* = 10.5, 3.7 Hz, 1H, 2'-H), 3.63 (m, 3H, linker-1H, 3''-H, 2''-H), 3.56 (d, *J* = 2.6, 1H, 4'-H), 3.53 (s, 1H, 4-H), 3.42 (m, 1H, linker-1H), 3.32 (m, 2H, linker-2H), 3.23 (td, *J* = 9.7, 4.8 Hz, 1H, 5''-H), 1.83 (m, 2H, linker-2H), 1.21 (m, 6H, 6-CH_3_, 6'-CH_3_); ^13^C NMR (100 MHz, CDCl_3_) δ = 138.4, 137.9, 137.3, 136.8, 129.0, 128.6, 128.52, 128.49, 128.4, 128.3, 128.2, 128.1, 128.0, 127.94, 127.9, 127.82, 127.78, 127.7, 127.4, 126.1, 101.6, 99.9, 98.2, 98.1, 79.9, 78.4, 77.22, 77.17, 76.9, 76.5, 76.4, 75.7, 75.3, 75.2, 73.0, 68.4, 67.8, 67.5, 67.3, 66.9, 66.0, 63.6, 60.3, 58.5, 16.8.

*N*-Benzyl-*N*-benzyloxycarbonyl-3-aminopropyl 4-*O*-benzyl-3-*O*-(4-*O*-benzyl-3-*O*-[2-acetamido-4,6-*O*-benzylidene-3-*O*-benzyl-2-deoxy-β-D-mannopyranosyl]-2-acetamido-2-deoxy-α-L-fucopyranosyl)-2-acetamido-2-deoxy-α-D-fucopyranoside (**21**)





To a solution of **20** (12.2 mg, 10 μmol) in pyridine (1 mL) were added water (289 μL, 16.05 mmol), Et_3_N (62 μL, 0.45 mmol) and propane-1,3-dithiol (60 μL, 0.60 mmol). The reaction mixture was stirred at room temperature for 4 h. Upon reaction completion, the reaction mixture was concentrated and the crude amine product was used in next step without further purification.

To a solution of above aminosugar in MeOH (0.5 mL) was added Ac_2_O (12 μL, 0.12 mmol) under argon. The reaction mixture was stirred at room temperature for 18 h. Upon reaction completion, the reaction mixture was concentrated and the residue was purified by silica gel column chromatography (DCM : MeOH 40 : 1 *v*/*v*) to afford product **21** as yellow syrup (8.3 mg, 6.7 μmol, 67% over two steps). IR ν_max_ (film) 3326, 2872, 1665, 1530, 1453, 1370, 1087, 1047, 734, 697, 576 cm^-1^; ^1^H NMR (400 MHz, CDCl_3_) δ = 7.60-7.12 (m, 30H, 6Ph), 7.02 (d, *J* = 9.3 Hz, 1H, 2-NH), 6.25 (d, *J* = 9.6 Hz, 1H, 2'-NH), 6.16 (d, *J* = 8.4 Hz, 1H, 2''-NH), 5.56 (s, 1H, PhCH), 5.24-5.05 (m, 2H, Bn-2H), 4.94-4.81 (m, 3H, Bn-2H, 1'-H), 4.77 (m, 4H, Bn-2H, 2-H, 2''-H), 4.70-4.55 (m, 4H, Bn-2H, 2'-H, 1-H), 4.51 (d, *J* = 2.1 Hz, 1H, 1''-H), 4.46 (d, *J* = 11.3 Hz, 1H, Bn-1H), 4.37 (d, *J* = 15.9 Hz, 1H, Bn-1H), 4.22 (dd, *J* = 10.8, 4.7 Hz, 1H, 6''-CH_a_), 4.00-3.89 (m, 2H, 5-H, 3-H), 3.84 (d, *J* = 6.7 Hz, 1H, 5'-H), 3.79-3.57 (m, 6H, linker-2H, 6''-CH_b_, 4''-H, 3''-H, 3-H), 3.46 (dd, *J* = 5.8, 2.6 Hz, 2H, 4-H, 4'-H), 3.37-3.10 (m, 3H, linker-2H, 5''-H), 2.12 (s, 3H, CH_3_CO), 2.01 (s, 6H, 2CH_3_CO), 1.73 (m, 2H, linker-2H), 1.26 (d, *J* = 6.5 Hz, 3H, 6-CH_3_), 1.22 (d, *J* = 6.4 Hz, 3H, 6'-CH_3_); ^13^C NMR (100 MHz, CDCl_3_) δ = 172.0, 171.4, 156.5, 138.6, 138.5, 138.2, 137.4, 137.3, 136.4, 128.9, 128.7, 128.50, 128.47, 128.3, 128.24, 128.19, 127.9, 127.8, 127.7, 127.5, 127.2, 127.1, 126.1, 101.5, 100.4, 98.8, 97.9, 80.6, 79.5, 78.4, 76.1, 75.4, 75.1, 71.6, 68.6, 67.5, 67.0, 51.0, 49.7, 48.8, 47.7, 24.1, 23.2, 16.9, 16.8.

Table S1 Comparison of ^1^H and ^13^C NMR chemical shifts between isolated polysaccharide and synthetic 1

| Residue | | Chemical shifts (ppm) | | | | | | | |
| --- | --- | --- | --- | --- | --- | --- | --- | --- | --- |
|  |  | H1/C1 | H2/C2 | H3/C3 | H4/C4 | H5/C5 | H6/C6 | NAc | OAc |
| →3)-β-D-  ManpNAcA  (4OAc)  -(1→ | isolated | 4.96/  96.0 | 4.50/  54.1 | 4.15/  75.6 | 5.17/  71.9 | 3.79/  75.7 |  | 2.07/  23.8 | 2.15/  21.6 |
|  | synthetic | 5.02/  95.1 | 4.59/  53.0 | 4.13/  66.9 | 5.09/  70.1 | 3.98/  74.1 |  | 2.11/  22.3 | 2.18/  20.3 |
| →3)-α-L-  FucpNAc  -(1→ | isolated | 4.98/  100.2 | 4.19/  49.0 | 4.19/  74.3 | 4.04/  69.1 | 4.05/  68.3 | 1.26/  16.7 | 1.96/  23.5 |  |
|  | synthetic | 5.07/  98.9 | 4.23/  47.6 | 4.08/  67.6 | 4.23/  73.1 | 4.13/  69.5 | 1.28/  15.5 | 2.03/  21.9 |  |
| →3)-α-D-  FucpNAc  -(1→ | isolated | 4.95/  100.2 | 4.27/  49.9 | 3.77/  72.6 | 3.75/  75.0 | 4.28/  68.2 | 1.26/  16.9 | 2.07/  23.3 |  |
|  | synthetic | 4.83/  97.2 | 4.33/  48.5 | 4.00/  73.3 | 3.85/  71.1 | 4.13/  66.5 | 1.27/  15.3 | 2.11/  21.9 |  |


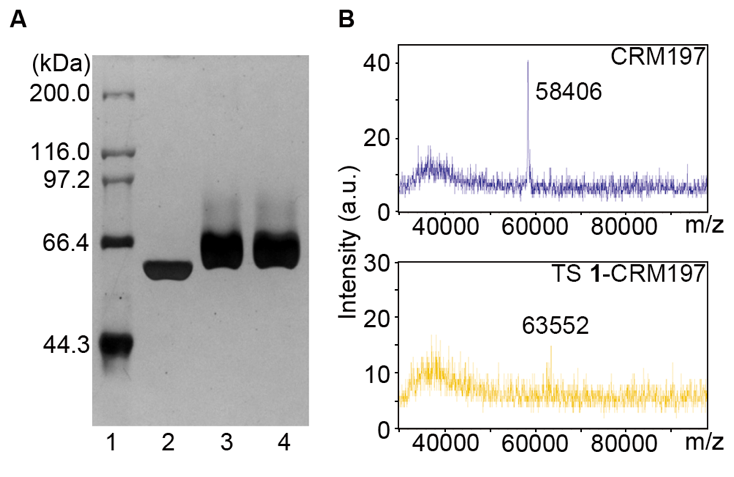


**Figure S1. Characterization of the glycoconjugate. (A)** The glycoconjugate resolved on SDS-PAGE and stained with silver. 1: marker, 2: CRM197, 3: trisaccharide **1**-CRM197 in H_2_O, 4: trisaccharide **1**-CRM197 in PBS. **(B)** MALDI-TOF/TOF-MS analysis of the average molecular of glycoconjugate trisaccharide **1**-CRM197.


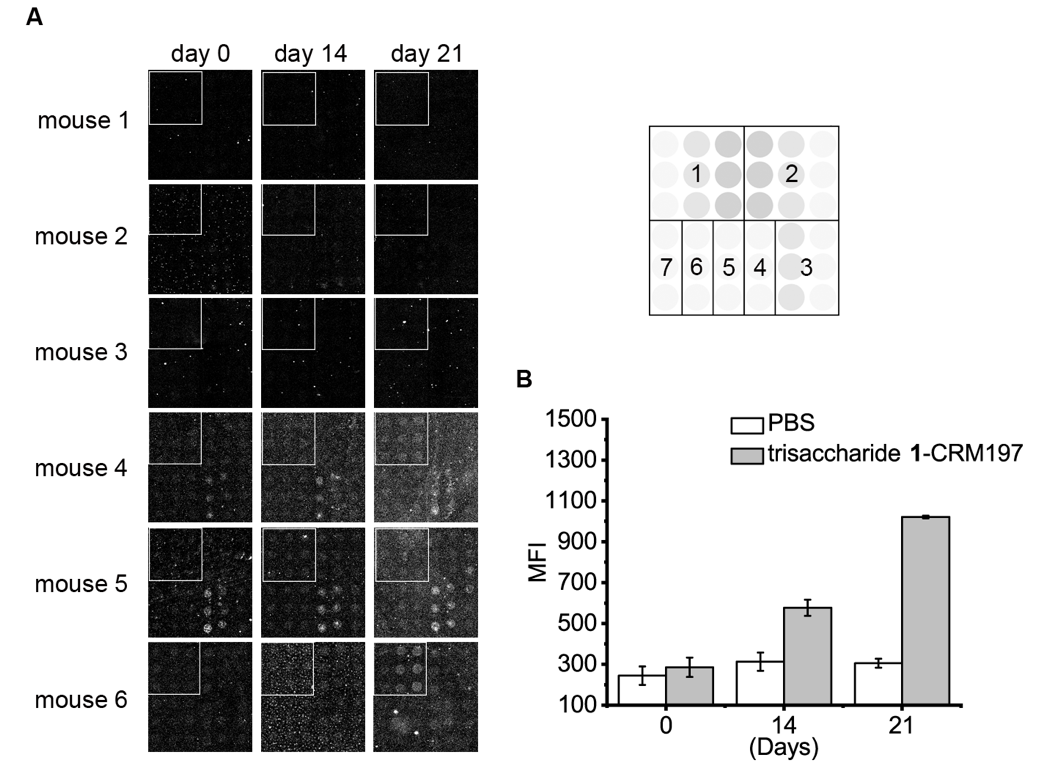


**Figure S2. Glycan microarray analysis of the mice sera. (A)** IgG antibodies were detected with secondary anti-mouse IgG Alexa Fluor 488 antibody. The microarray printing pattern is shown to the right of the scan. 1: trisaccharide **1** was spotted at 0.1, 0.5 and 1 mM; 2: linker was spotted at 1, 0.5 and 0.1 μM. 3: CRM197 was spotted at 0.1 and 0.05μM. 4: *E. coli* O55: B5 LPS was spotted at 0.2mg/mL. 5: buffer, 50mM sodium phosphate, pH 8.5. 6,7 was spotted at 0.5mM 6: *P. shigelloides* serotype 51 O-antigen trisaccharide. 7: α-1-6-glucose trisaccharide. Mouse 1-3 were immunized with PBS. Mouse 4-6 were immunized with trisaccharide **1**-CRM197. **(B)** Quantification of mean fluorescence intensities of sera of PBS group and trisaccharide **1**-CRM197 group against trisaccharide **1**. Error bars represent SD of three spots of two separate arrays, respectively.


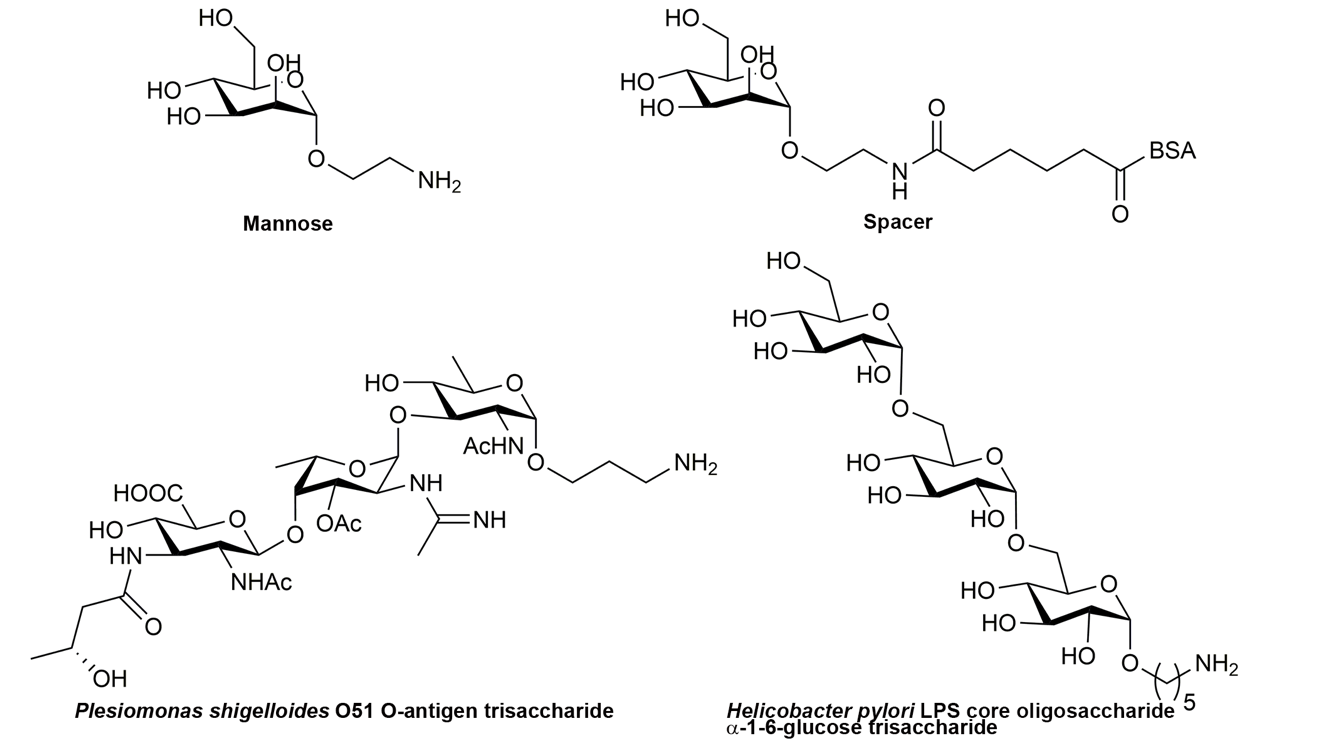


**Figure S3. Control glycan structures for microarray analysis.**

**
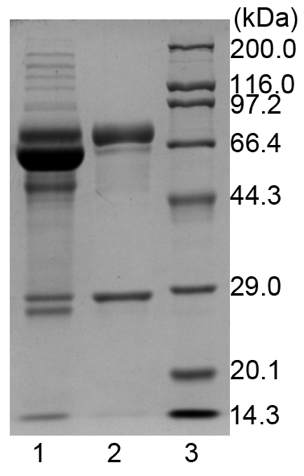
**

**Figure S4. The SDS-PAGE analysis of the purity of the monoclonal antibody.** The unpurified ascetic fluid and purified ascetic fluid resolved on SDS-PAGE and stained with Coomassie Blue.1：unpurified ascitic fluid, 2: purified ascitic fluid, 3: marker. The center lane contains the heavy chain (upper band) and light chains (lower band).

**NMR spectra of synthetic products**

^1^H NMR (CDCl_3_, 400 MHz) of compound **S1**


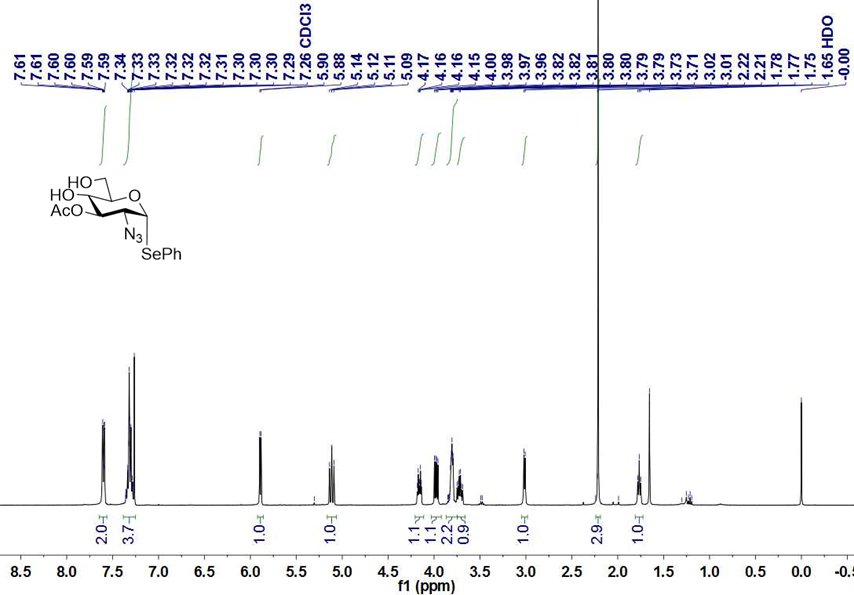


^13^C NMR (CDCl_3_, 100 MHz) of compound **S1**


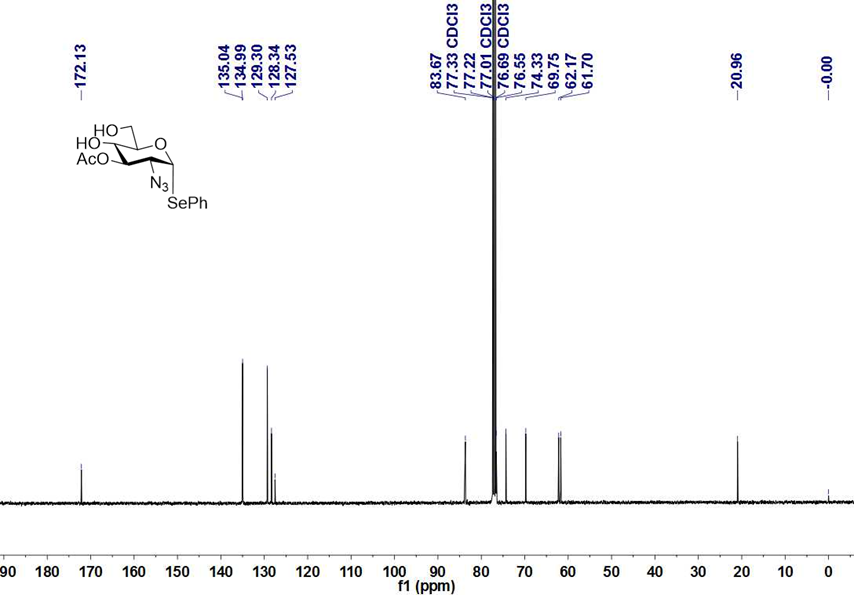


^1^H NMR (CDCl_3_, 400 MHz) of compound **8**


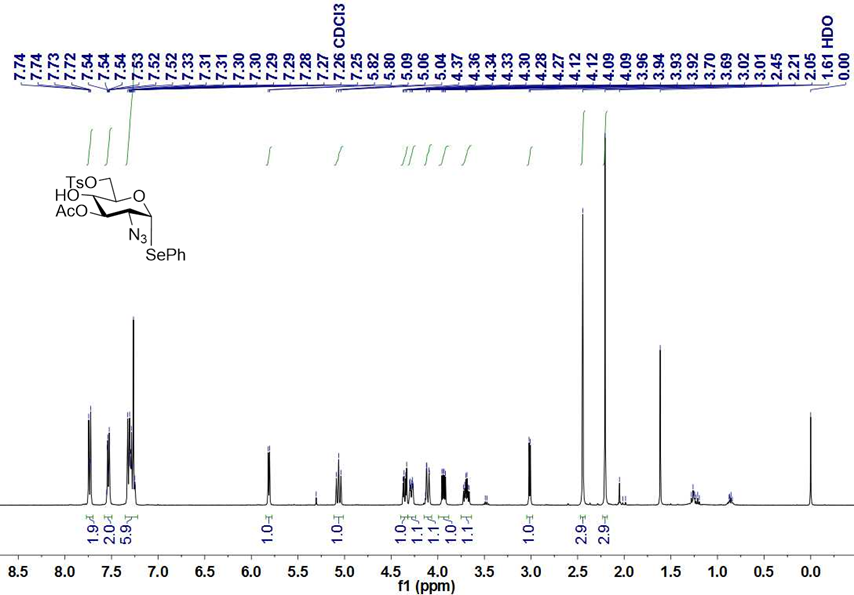


^1^H NMR (CDCl_3_, 400 MHz) of compound **S2**


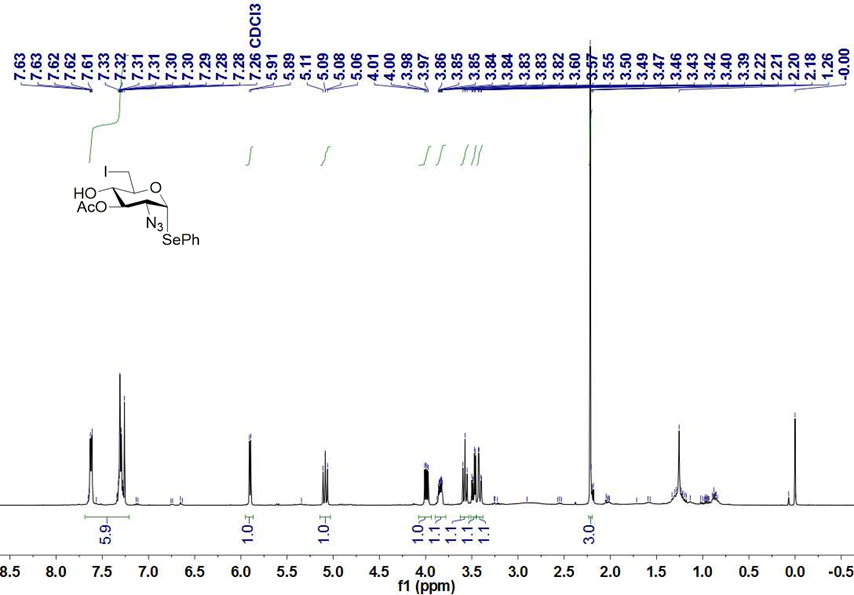


^13^C NMR (CDCl_3_, 100 MHz) of compound **S2**


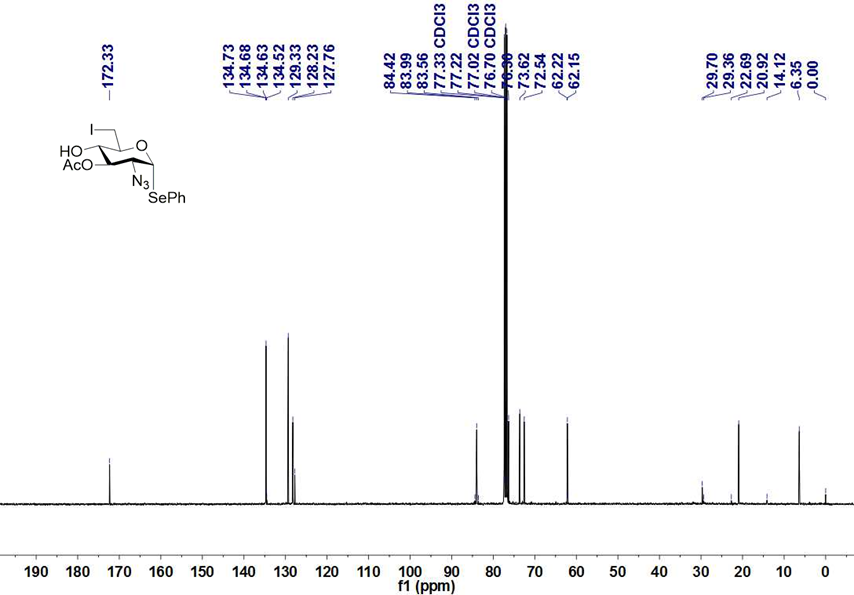


^1^H NMR (CDCl_3_, 400 MHz) of compound **9**


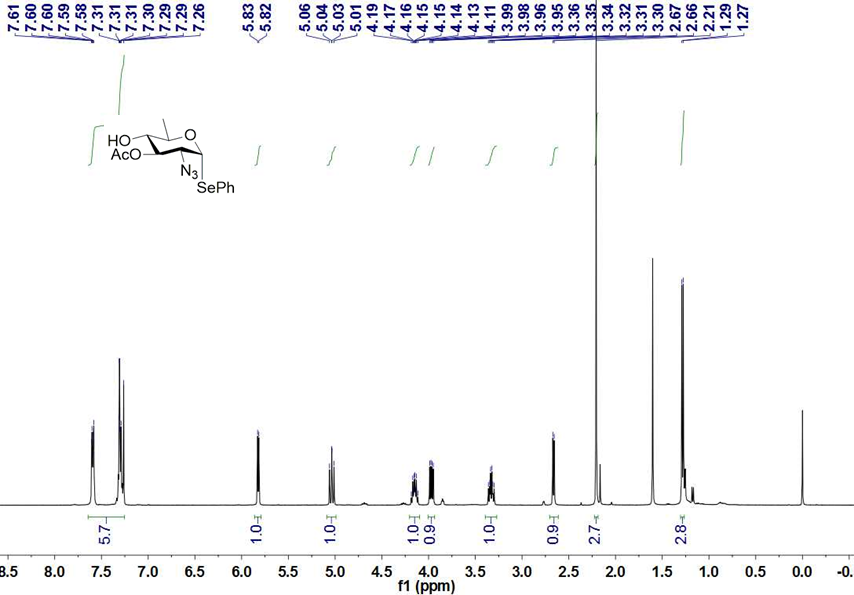


^13^C NMR (CDCl_3_, 100 MHz) of compound **9**


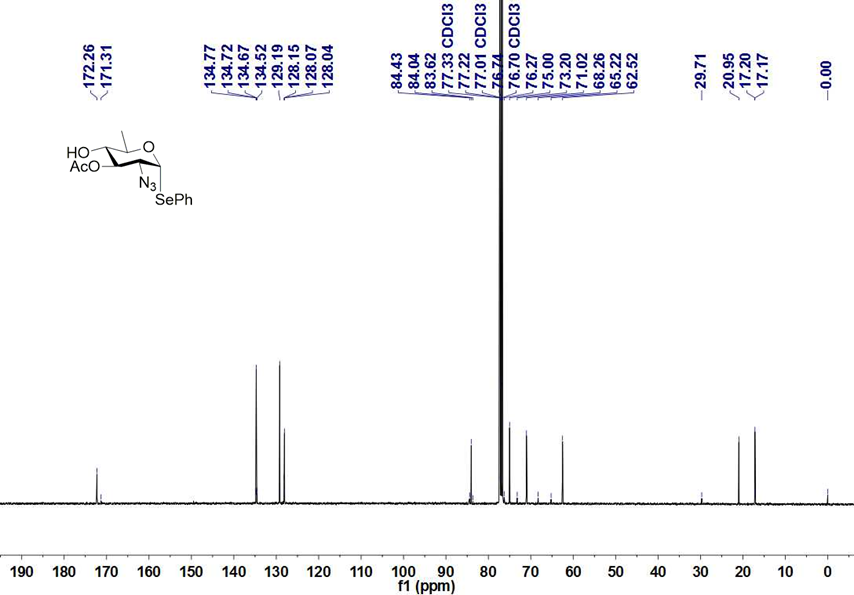


^1^H NMR (CDCl_3_, 400 MHz) of compound **S3**


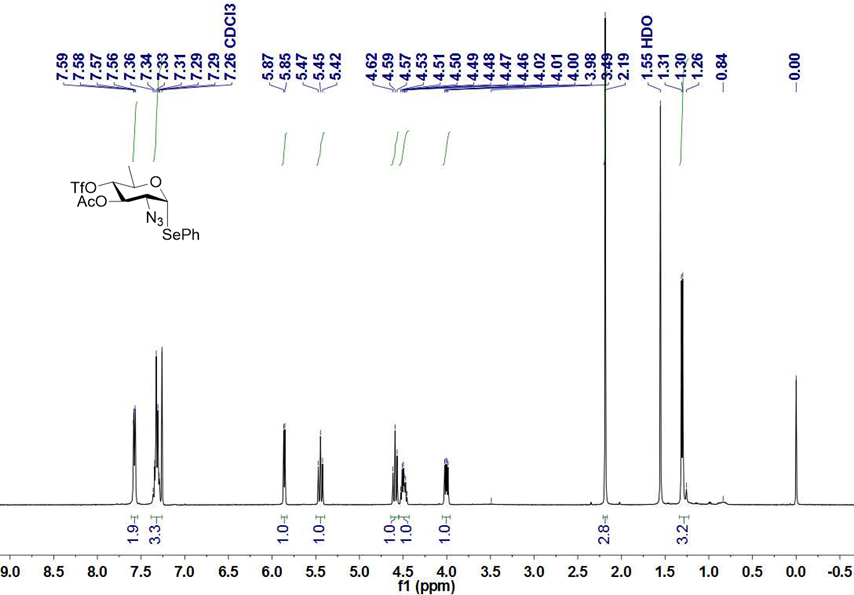


^13^C NMR (CDCl_3_, 100 MHz) of compound **S3**


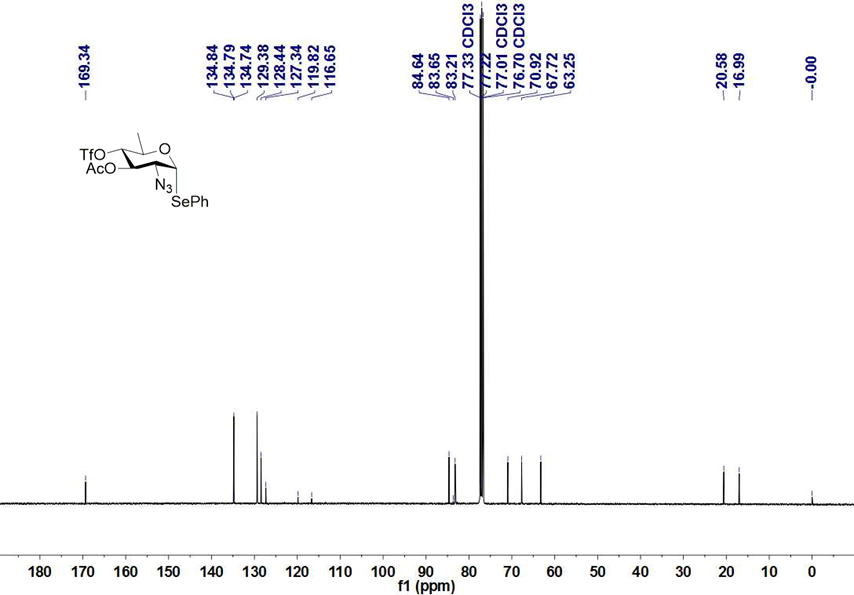


^1^H NMR (CDCl_3_, 400 MHz) of compound **10**


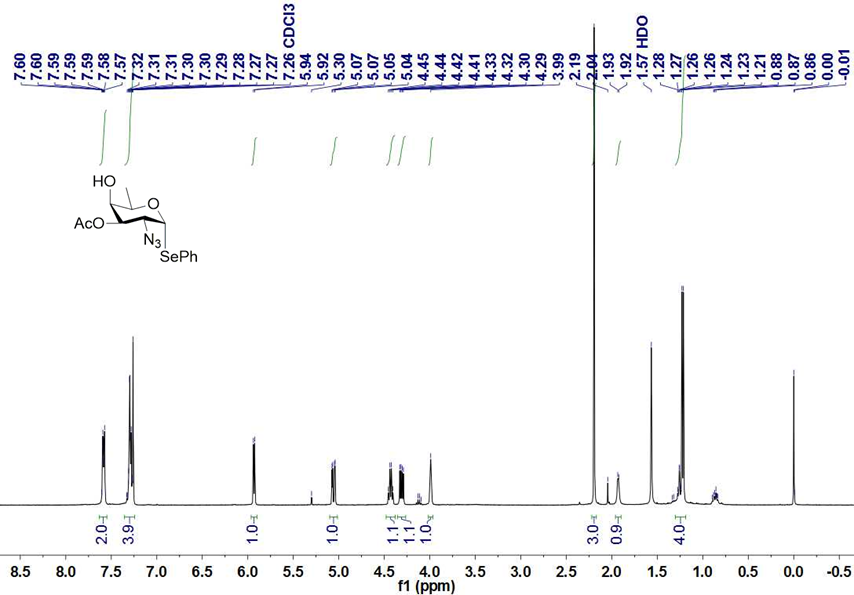


^1^H NMR (CDCl_3_, 400 MHz) of compound **11**


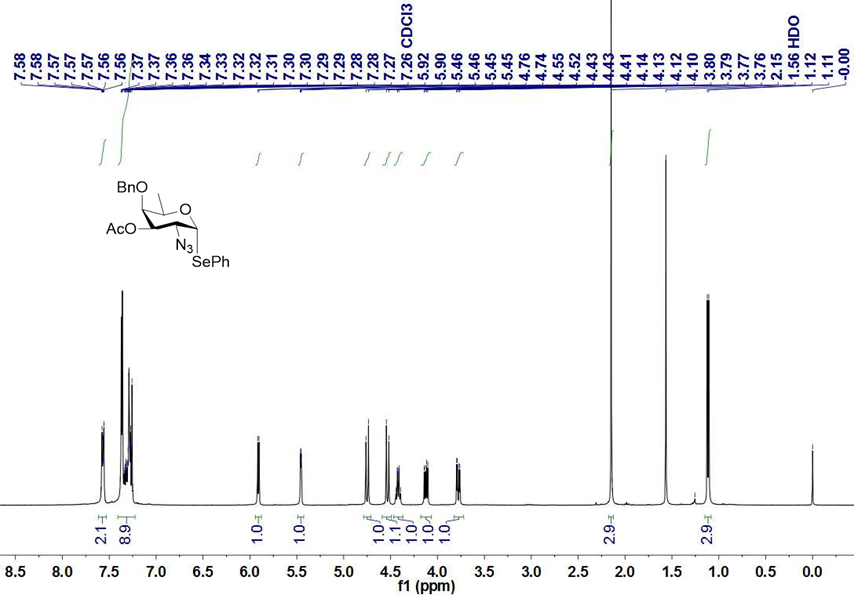


^13^C NMR (CDCl_3_, 100 MHz) of compound **11**


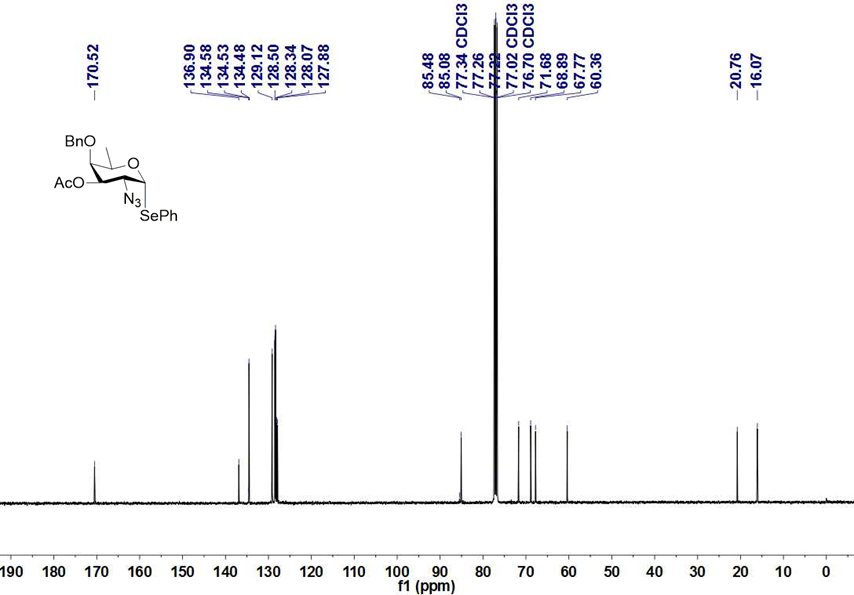


^1^H NMR (CDCl_3_, 400 MHz) of compound **13**


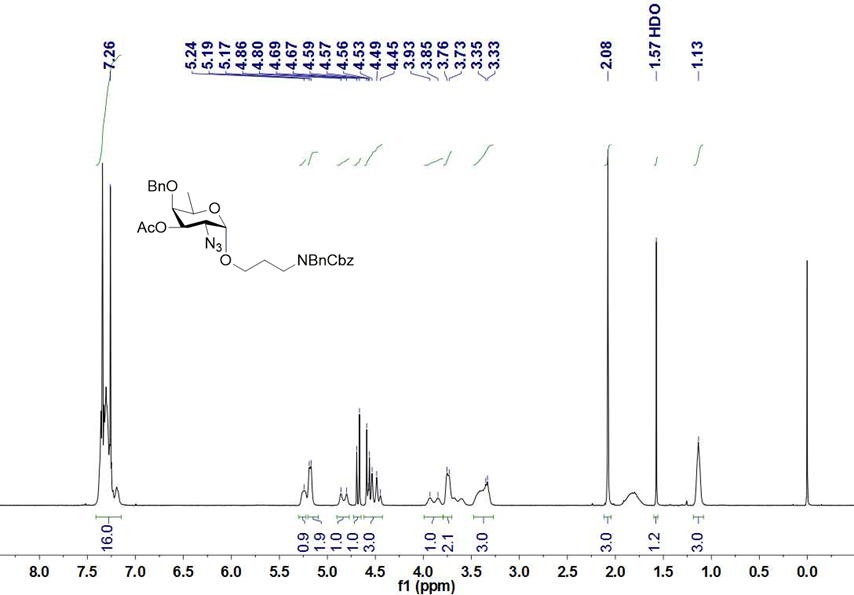


^13^C NMR (CDCl_3_, 100 MHz) of compound **13**


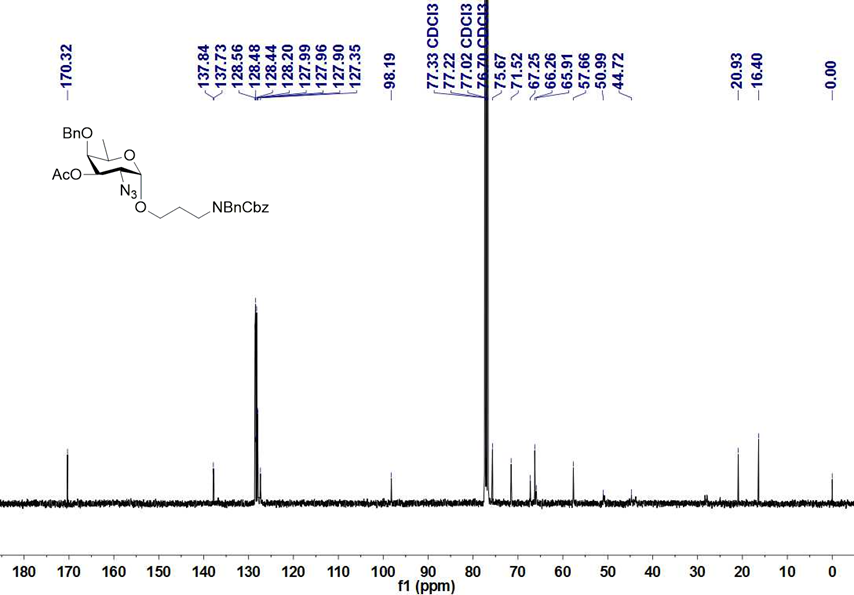


^1^H NMR (CDCl_3_, 400 MHz) of compound **4**


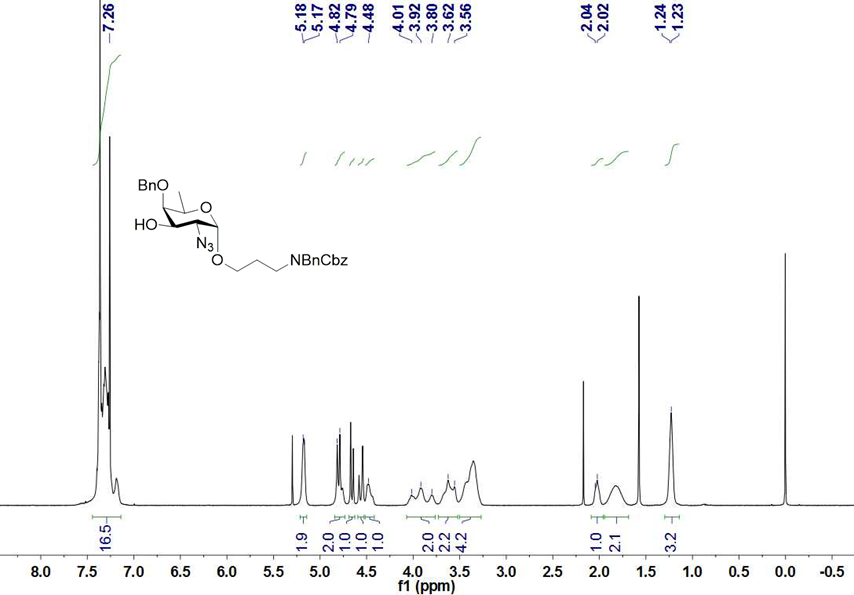


^1^H NMR (CDCl_3_, 400 MHz) of compound **15**

**
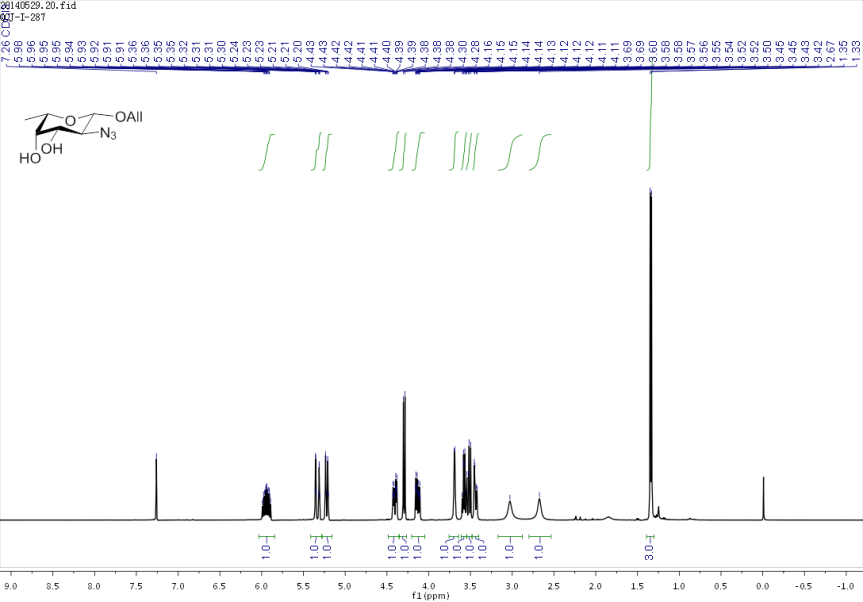
**

^13^C NMR (CDCl_3_, 100 MHz) of compound **15**

**
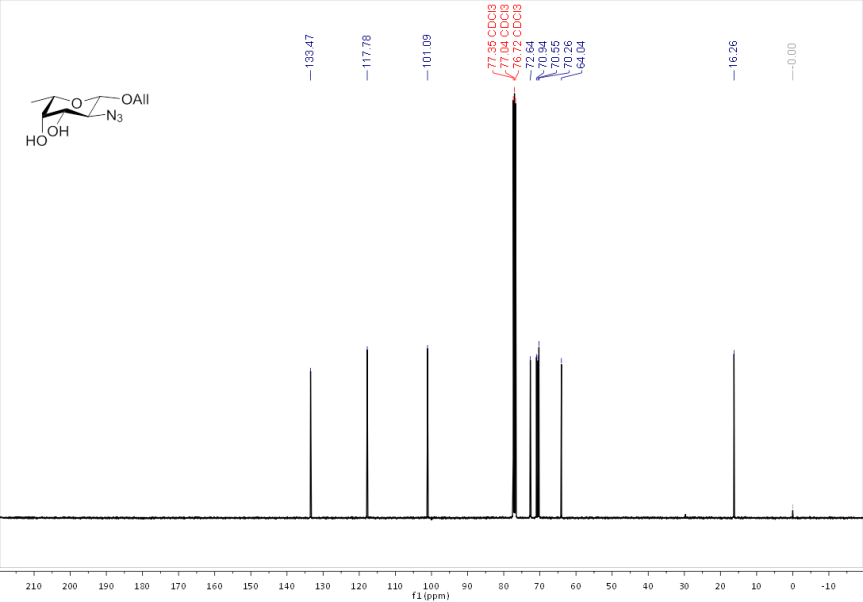
**

^1^H NMR (CDCl_3_, 400 MHz) of compound **16**


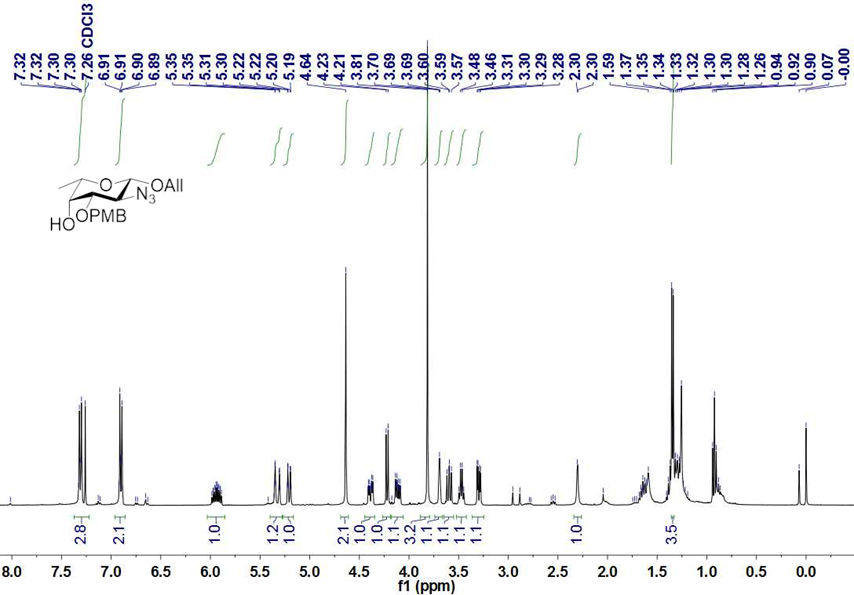


^1^H NMR (CDCl_3_, 400 MHz) of compound **17**


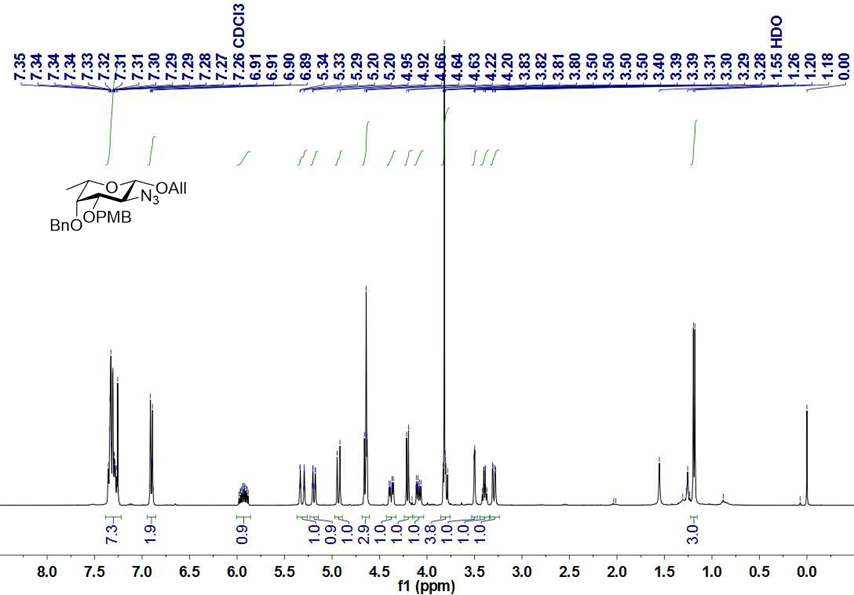


^13^C NMR (CDCl_3_, 100 MHz) of compound **17**


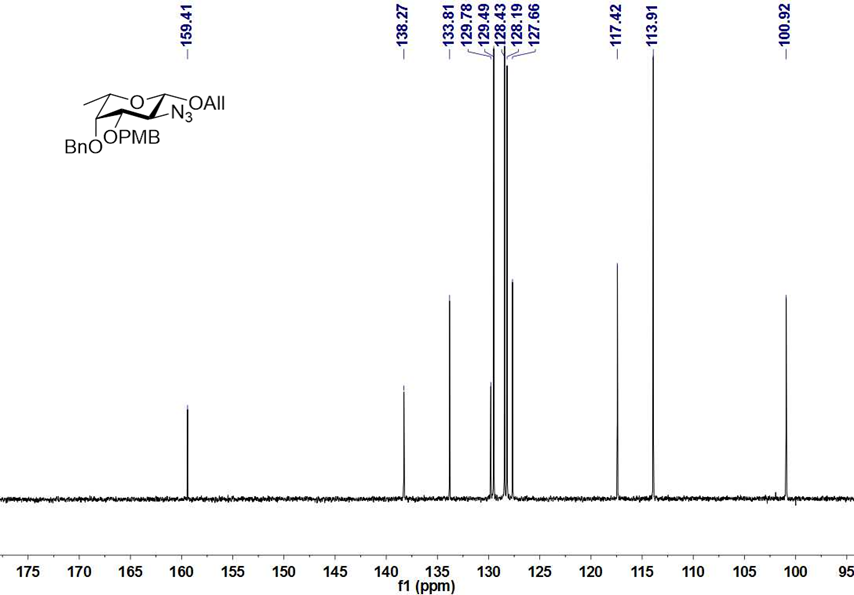


^1^H NMR (CDCl_3_, 400 MHz) of compound **6**


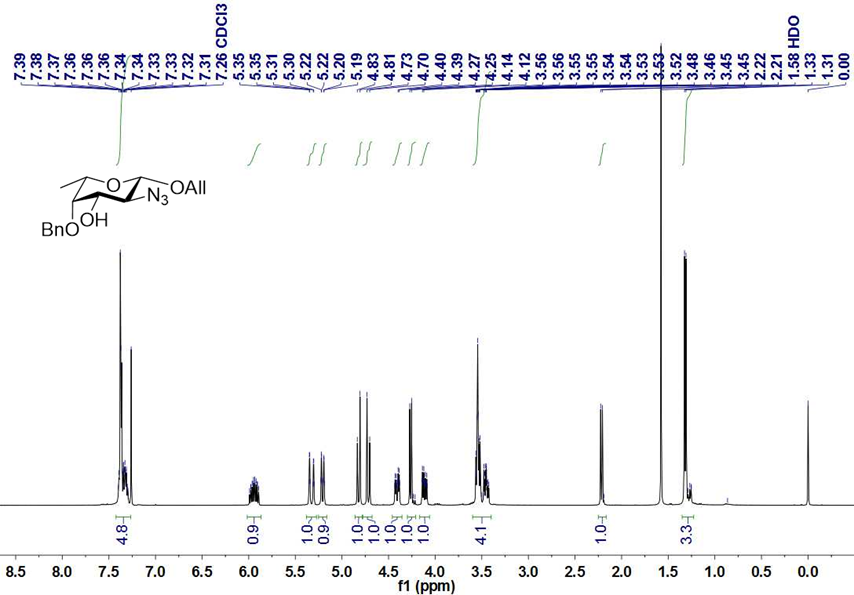


^13^C NMR (CDCl_3_, 100 MHz) of compound **6**


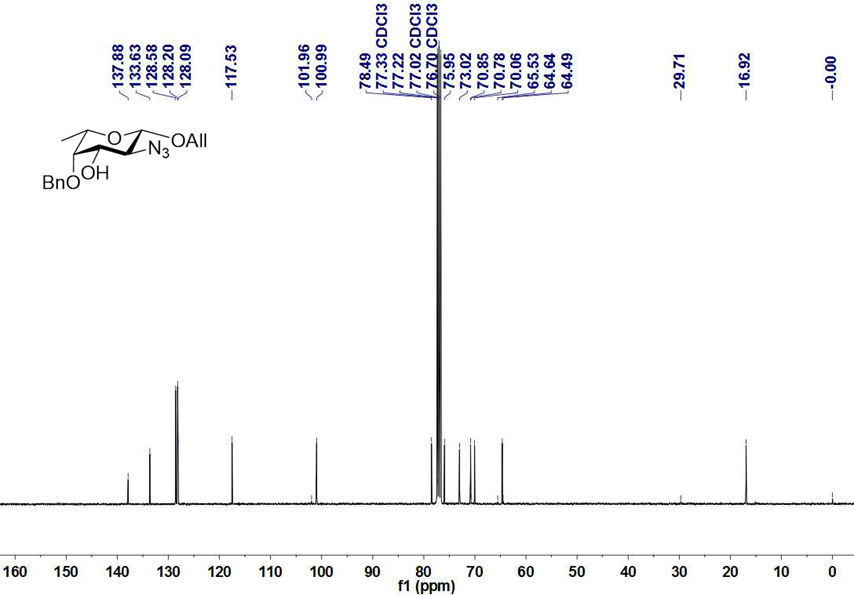


^1^H NMR (CDCl_3_, 400 MHz) of compound **18**


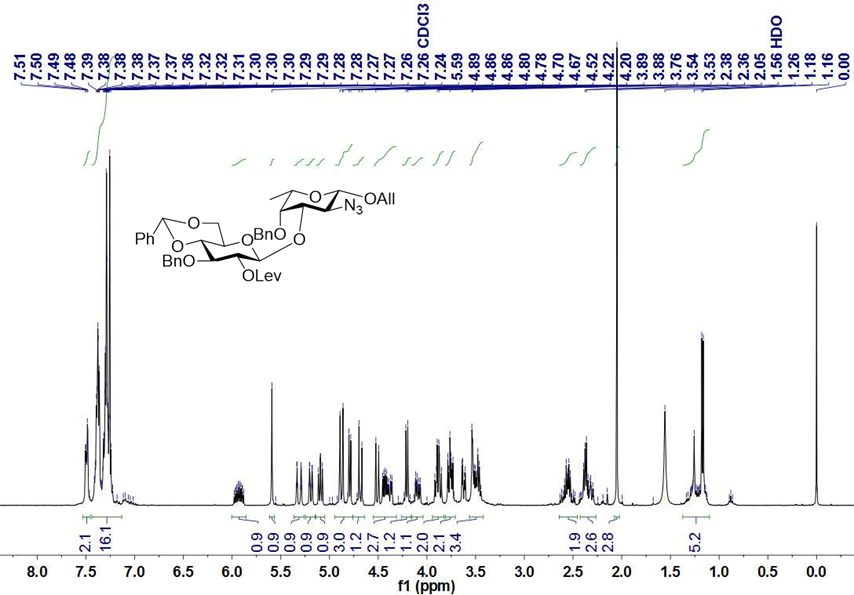


^1^H NMR (CDCl_3_, 400 MHz) of compound **S4**


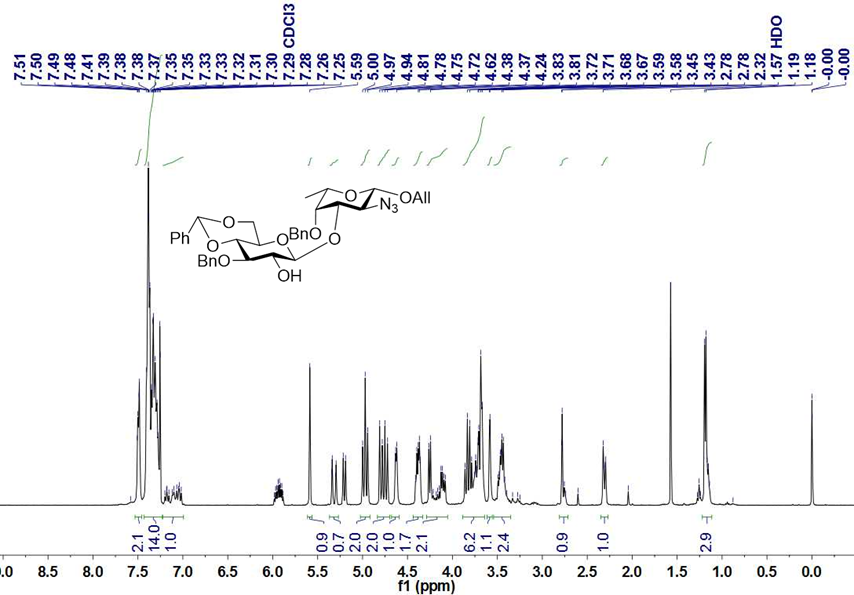


^13^C NMR (CDCl_3_, 100 MHz) of compound **S4**


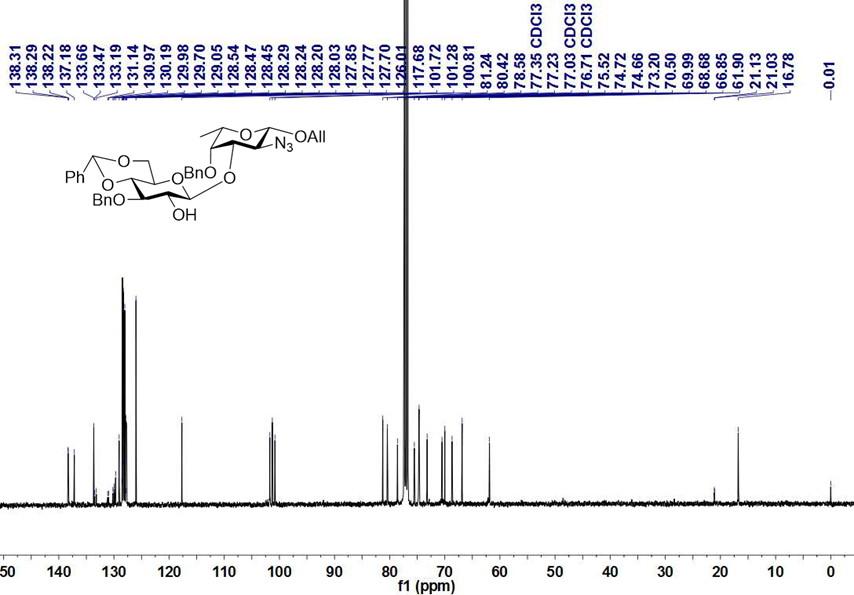


^1^H NMR (CDCl_3_, 400 MHz) of compound **19**


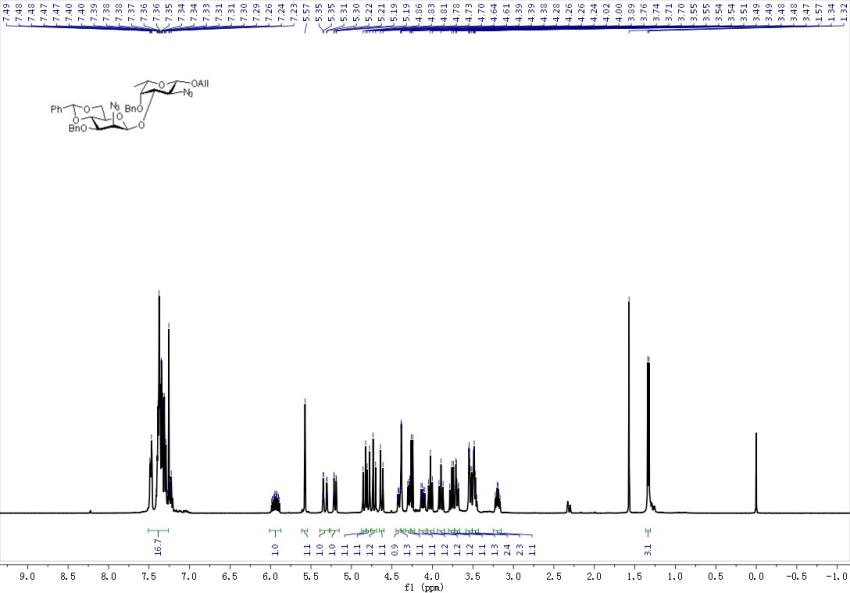


^13^C NMR (CDCl_3_, 100 MHz) of compound **19**


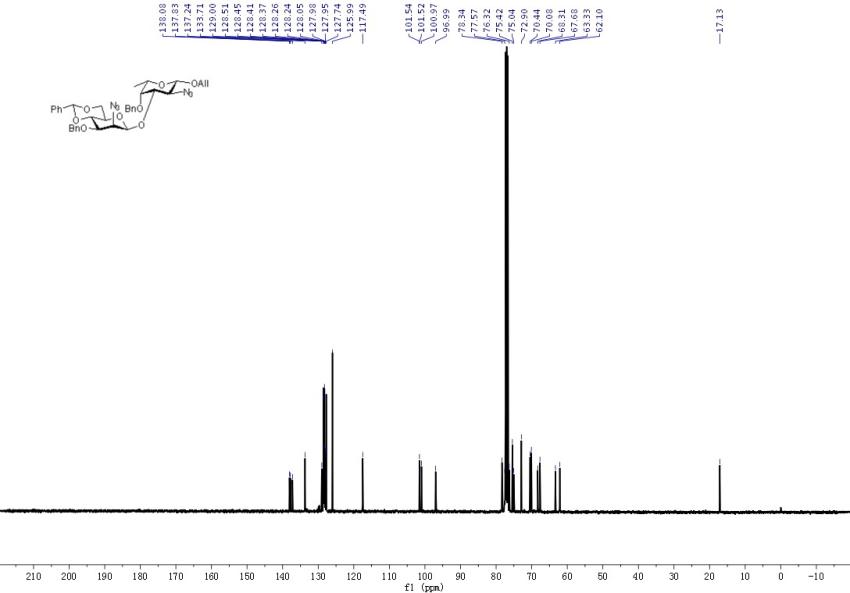


^1^H NMR (CDCl_3_, 400 MHz) of compound **20**


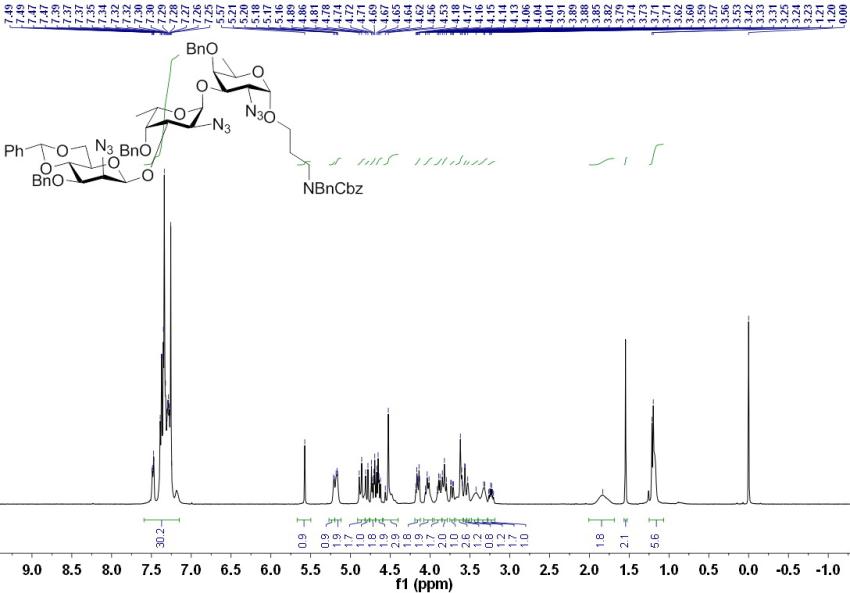


^13^C NMR (CDCl_3_, 100 MHz) of compound **20**


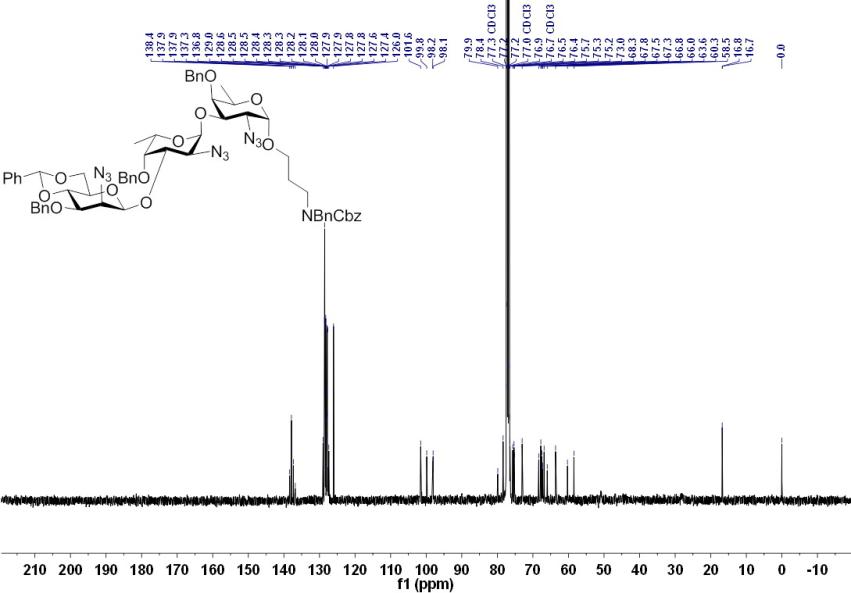


^1^H-^1^H COSY (CDCl_3_, 400 MHz) of compound **20**


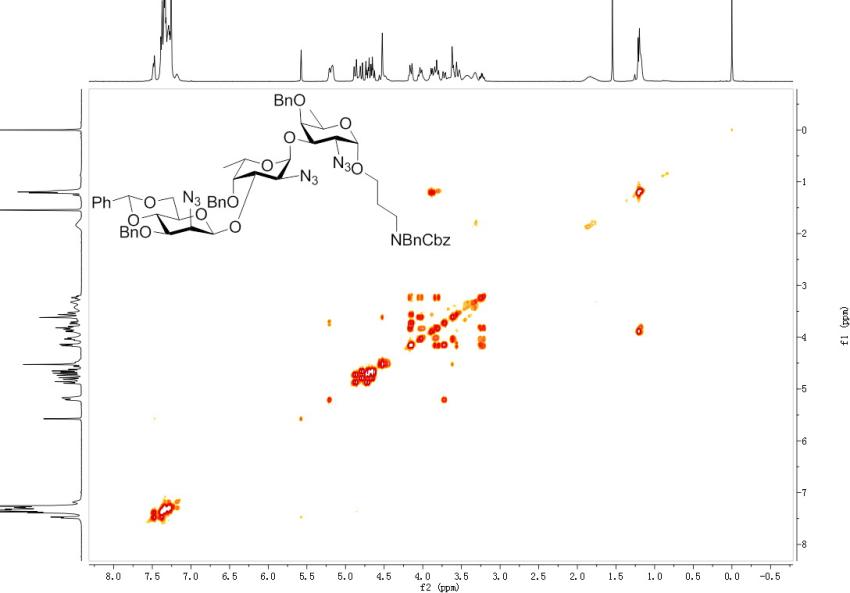


^1^H NMR (CDCl_3_, 400 MHz) of compound **21**


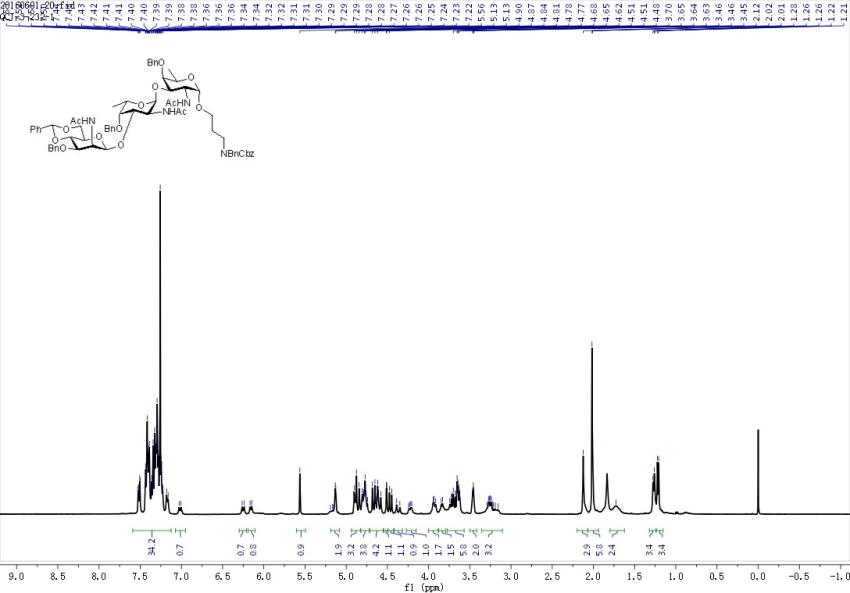


^13^C NMR (CDCl_3_, 100 MHz) of compound **21**


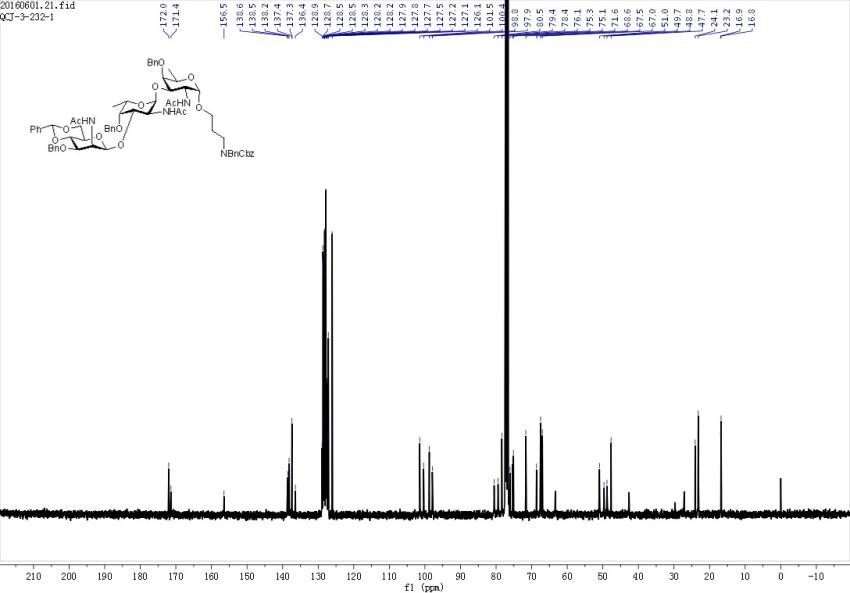


^1^H NMR (CDCl_3_, 400 MHz) of compound **2**


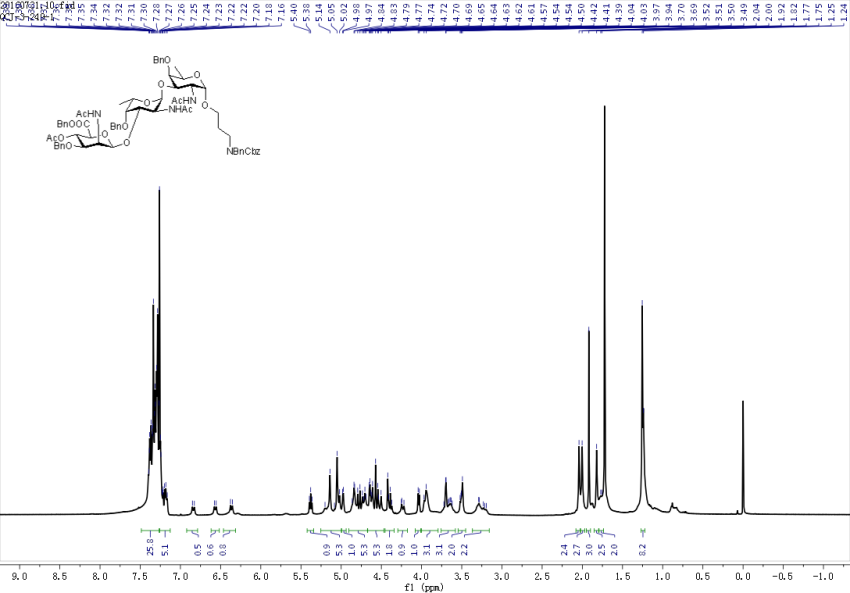


^13^C NMR (CDCl_3_, 100 MHz) of compound **2**


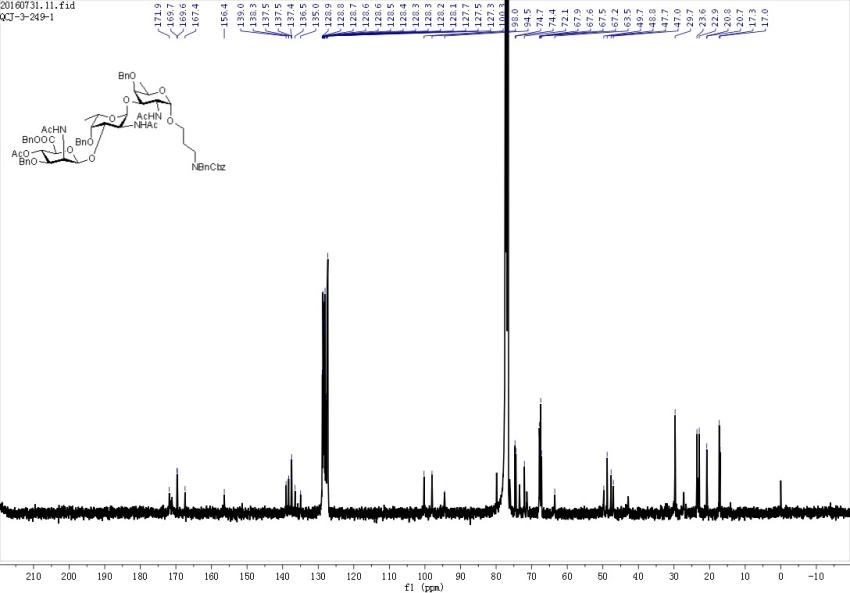


^1^H-^1^H COSY (CDCl_3_, 400 MHz) of compound **2**


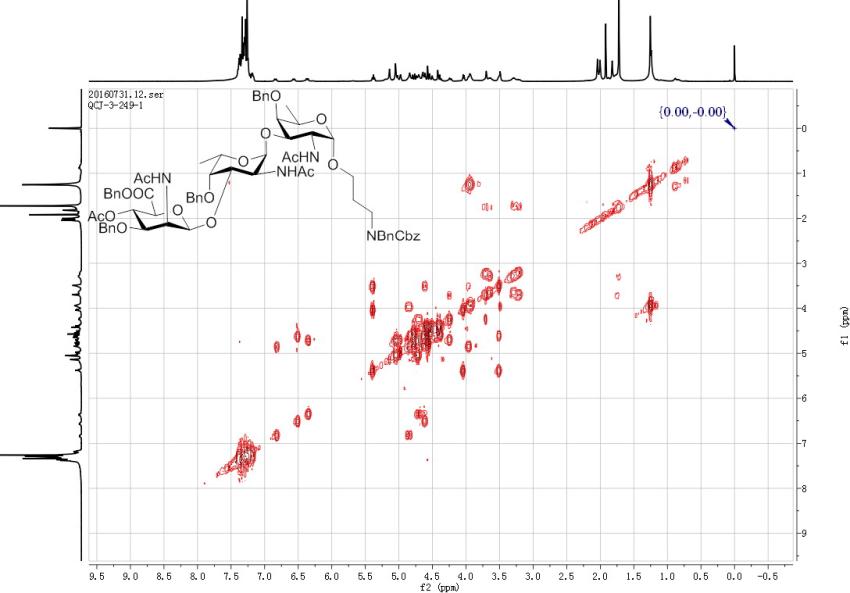


^1^H-^13^C HSQC (CDCl_3_, 400 MHz) of compound **2**


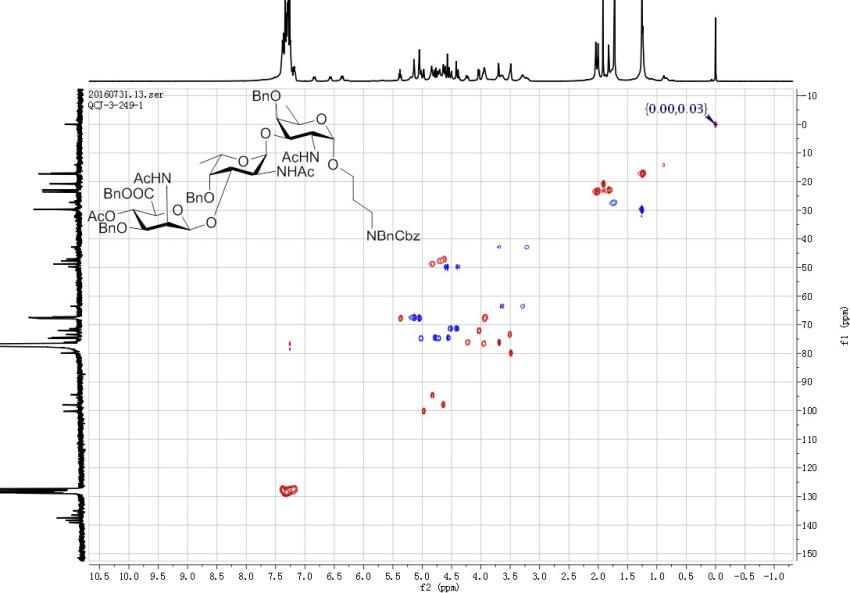


^1^H NMR (D_2_O, 400 MHz) of compound **1**


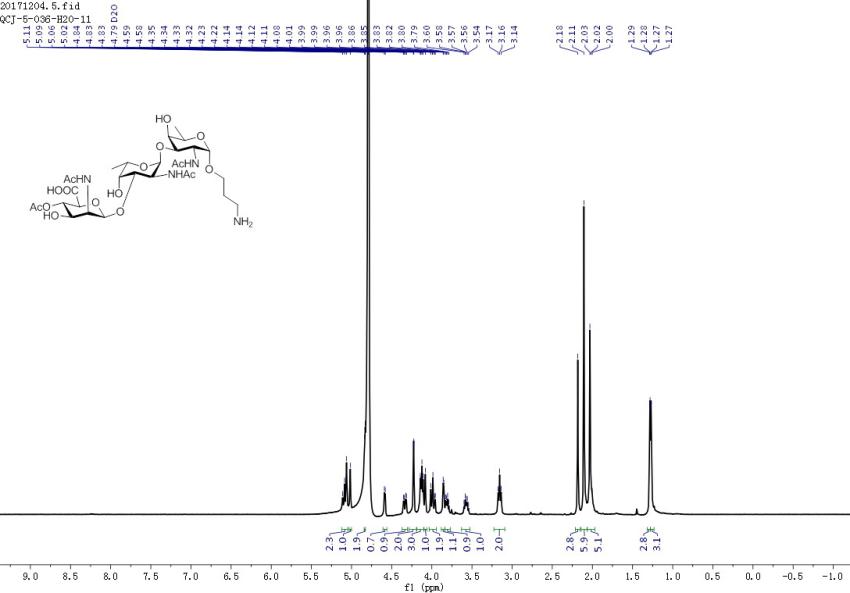


^13^C NMR (D_2_O, 100 MHz) of compound **1**


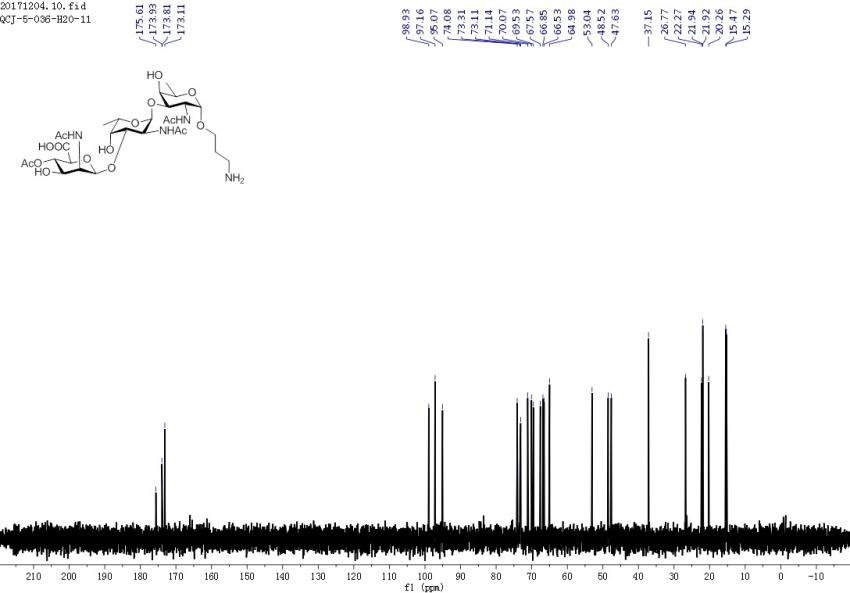


^1^H-^1^H COSY (D_2_O, 400 MHz) of compound **1**


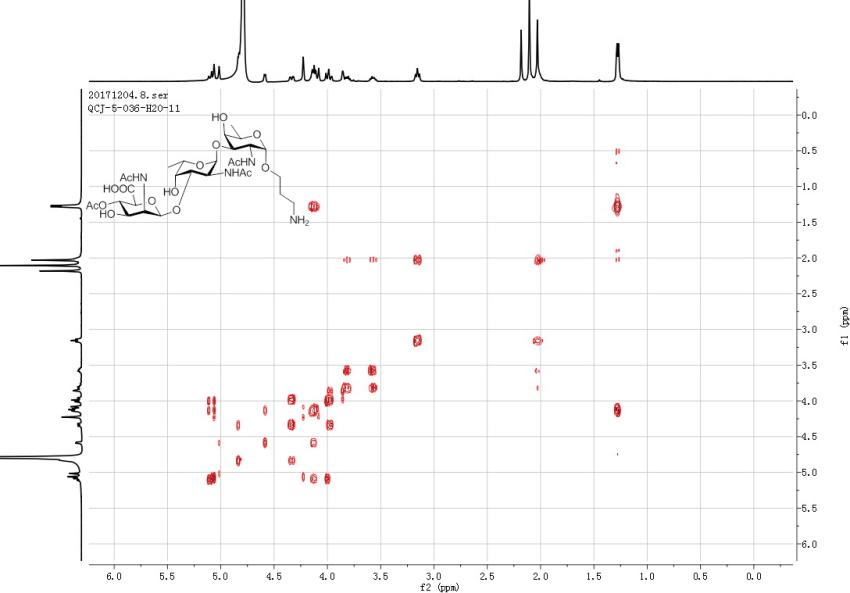


^1^H-^13^C HSQC (D_2_O, 400 MHz) of compound **1**


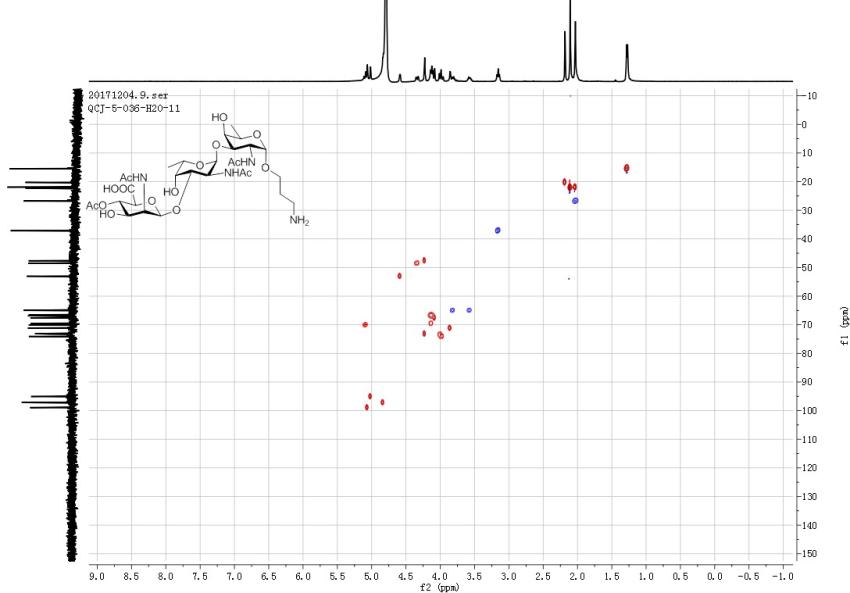

Supplement: Supplementary file 1 [file Table_1.DOCX]
